# Transcriptional analysis of lung fibroblasts identifies PIM1 signaling as a driver of aging-associated persistent fibrosis

**DOI:** 10.1172/jci.insight.153672

**Published:** 2022-03-22

**Authors:** Tho X. Pham, Jisu Lee, Jiazhen Guan, Nunzia Caporarello, Jeffrey A. Meridew, Dakota L. Jones, Qi Tan, Steven K. Huang, Daniel J. Tschumperlin, Giovanni Ligresti

**Affiliations:** 1 Department of Medicine, Boston University School of Medicine, Boston, Massachusetts, USA.; 2 Department of Physiology & Biomedical Engineering, Mayo Clinic, Rochester, Minnesota, USA.; 3 Department of Internal Medicine, University of Michigan Medical School, Ann Arbor, Michigan, USA.

**Keywords:** Aging, Pulmonology, Fibrosis

## Abstract

Idiopathic pulmonary fibrosis (IPF) is an aging-associated disease characterized by myofibroblast accumulation and progressive lung scarring. To identify transcriptional gene programs driving persistent lung fibrosis in aging, we performed RNA-Seq on lung fibroblasts isolated from young and aged mice during the early resolution phase after bleomycin injury. We discovered that, relative to injured young fibroblasts, injured aged fibroblasts exhibited a profibrotic state characterized by elevated expression of genes implicated in inflammation, matrix remodeling, and cell survival. We identified the proviral integration site for Moloney murine leukemia virus 1 (PIM1) and its target nuclear factor of activated T cells-1 (NFATc1) as putative drivers of the sustained profibrotic gene signatures in injured aged fibroblasts. PIM1 and NFATc1 transcripts were enriched in a pathogenic fibroblast population recently discovered in IPF lungs, and their protein expression was abundant in fibroblastic foci. Overexpression of PIM1 in normal human lung fibroblasts potentiated their fibrogenic activation, and this effect was attenuated by NFATc1 inhibition. Pharmacological inhibition of PIM1 attenuated IPF fibroblast activation and sensitized them to apoptotic stimuli. Interruption of PIM1 signaling in IPF lung explants ex vivo inhibited prosurvival gene expression and collagen secretion, suggesting that targeting this pathway may represent a therapeutic strategy to block IPF progression.

## Graphical abstract

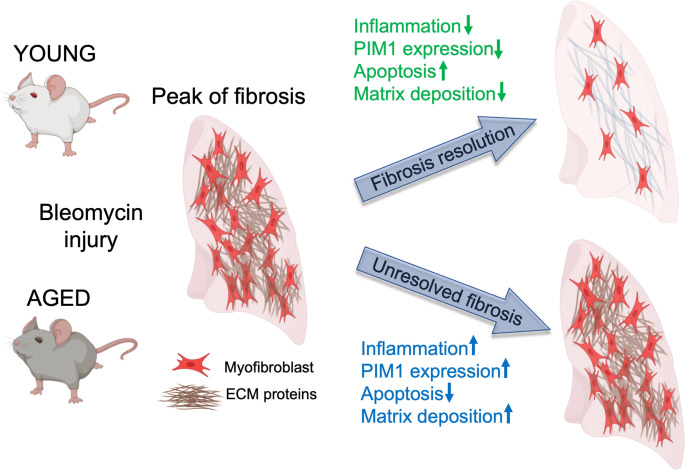

## Introduction

Idiopathic pulmonary fibrosis (IPF) is an aging-associated disease of the lung characterized by the accumulation of activated fibroblasts (myofibroblasts), excessive deposition of extracellular matrix (ECM) proteins, alveolar destruction, and lung stiffening (1, 2). Myofibroblasts are key cellular effectors of tissue remodeling in IPF lungs (3). Although these cells are transiently needed to orchestrate normal tissue repair, their pathogenic persistence and perpetuated activation leads to progressive lung fibrogenesis (4, 5). While multiple fibrogenic pathways have been found to drive aberrant matrix deposition (6), the transcriptional mechanisms responsible for maintaining persistent and self-sustaining myofibroblast activation remain largely unknown.

Recent single-cell RNA-Seq (scRNA-Seq) studies on mouse lungs following bleomycin challenge have revealed the remarkable heterogeneity of lung fibroblasts in both normal and fibrotic lungs (7, 8). Whereas these studies have offered a comprehensive characterization of different fibroblast transcriptomes in fibrotic mouse lungs, they have not provided specific insights into transcriptional programs governing lung fibrosis resolution and myofibroblast fate after injury. In this regard, we and others have recently characterized the transcriptome of lung fibroblasts at the peak of fibrosis and during the early resolution phase after bleomycin challenge in young mice and discovered that lung fibroblast activation in this model is transient and characterized by an acute elevation of profibrotic gene expression (14 days after bleomycin), followed by a gradual return to baseline during the initial resolution phase of fibrosis (30 days after bleomycin) (9–11). Notably, the acute profibrotic gene signature was similar to that of an aberrant myofibroblast population recently identified in IPF lungs (7), suggesting that transcriptional programs typically associated with the acute phase after injury may persist during sustained fibrosis, thereby promoting progressive accumulation of myofibroblasts and aberrant tissue remodeling.

While remarkable efforts have been dedicated to elucidating the origin of myofibroblasts in self-resolving mouse models of lung fibrosis, such as the bleomycin model in young mice (12), studies investigating myofibroblast persistence in progressive models of lung fibrosis are limited. Prior studies have identified NADPH oxidase 4 (NOX4), mouse double minute 4 homolog (MDM4), and the transcription factor myogenic differentiation 1 (MyoD) as factors that contributed to myofibroblast senescence and apoptosis resistance during persistent lung fibrosis in aged mice (11, 13–15). Consistent with these findings, we have recently shown that, similarly to IPF, activated fibroblasts accumulated and persisted in the fibrotic lungs of aged mice (16, 17), further suggesting that increased myofibroblast survival and/or dysfunctional apoptosis in aging may be responsible for the abnormal myofibroblast accumulation and for the sustained lung fibrosis.

To identify factors that may impair fibrosis resolution in advanced age, we performed a comparative RNA-Seq analysis on freshly isolated fibroblasts from the lungs of young and aged mice during the initial resolution phase of bleomycin-induced lung fibrosis (day 30 after bleomycin). We discovered that aged lung fibroblasts exhibited an increased inflammatory gene signature characterized by elevated expression of the proviral integration site for Moloney murine leukemia virus 1 (*Pim1*) relative to young lung fibroblasts. *Pim1* encodes for a protein kinase that phosphorylates and activates numerous nuclear proteins, including the transcription factor nuclear factor of activated T cells-1 (NFATc1), to promote cell proliferation and survival (18–20). Overexpression of PIM1 in normal human lung fibroblasts increased their fibrogenic activation, and this mechanism was attenuated by NFATc1 inhibition. Targeting PIM1 kinase using a small-molecule inhibitor blocked ECM protein synthesis and sensitized IPF-derived lung fibroblasts to apoptosis. Finally, inhibition of the PIM1 signaling pathway in organotypic cultures of human IPF lungs attenuated ECM remodeling and prosurvival gene expression, as well as collagen-I secretion. Together, our data strongly suggest that activation of PIM1/NFATc1 pathways in aging may perpetuate fibroblast activation and impair fibrosis resolution, and that its pharmacological inhibition may facilitate fibrosis resolution in IPF lungs.

## Results

### Lung fibroblasts from aged mice exhibit increased expression of inflammatory genes and reduced expression of ECM genes compared with young ones.

**Figure 1:**
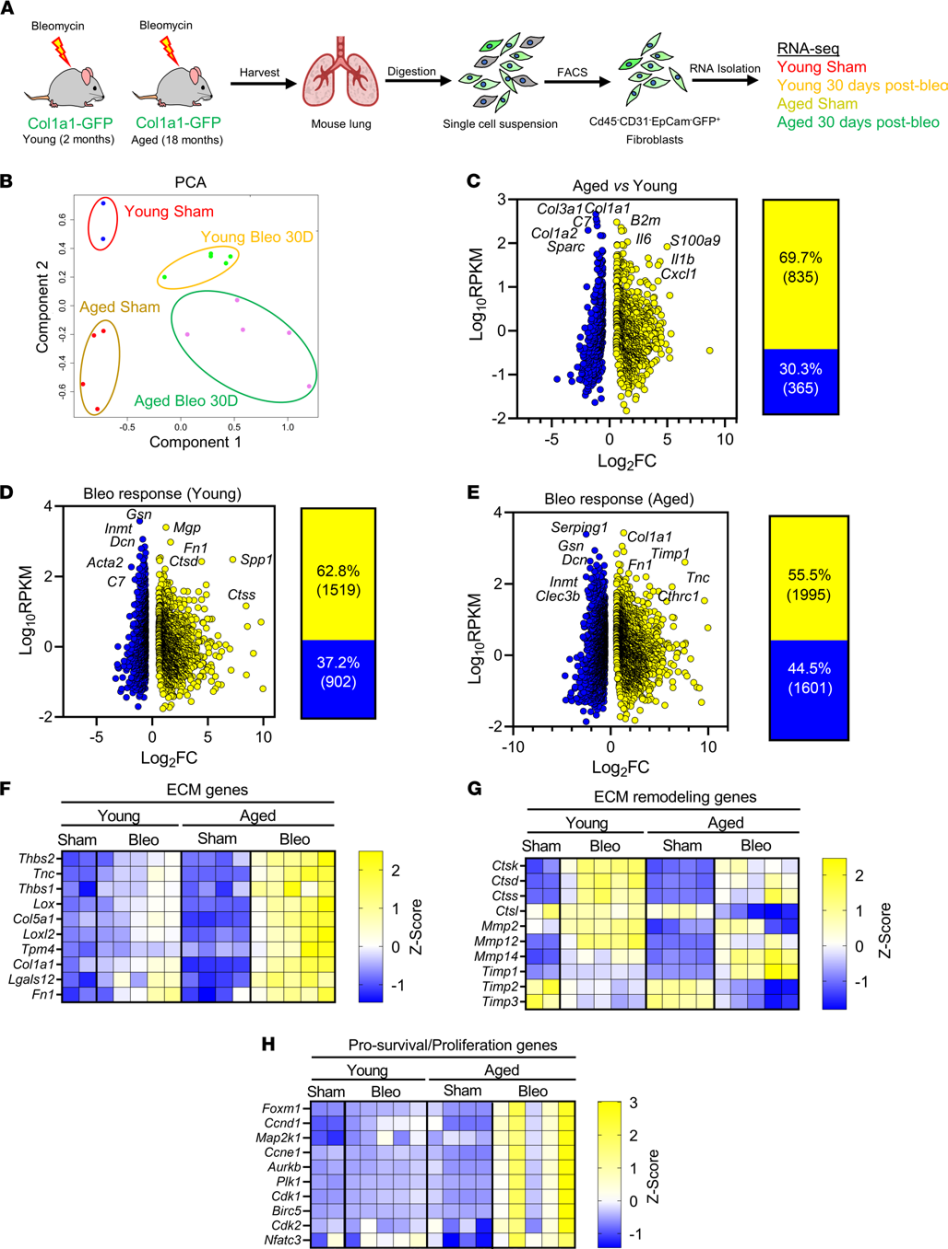
Impaired lung fibrosis resolution in aged mice is associated with increased inflammatory, ECM remodeling, and survival gene signatures in lung fibroblasts. (**A**) Young and aged Col1a1-GFP mice were exposed to saline or bleomycin and sacrificed after 30 days during early fibrosis resolution. Lungs were harvested, digested, and sorted by FACS for CD31^–^CD45^–^EpCAM^–^GFP^+^ lung fibroblasts and used for RNA-Seq analysis (young sham, *n* = 2; young bleo 30 days, *n* = 5; aged sham, *n* = 4; aged bleo 30 days, *n* = 5). (**B**) Principal components analysis (PCA) displaying clusters of samples from experimental groups and the similarity of their transcriptomes. (**C**–**E**) (Left) Plots of log_10_ (avg.RPKM) versus log_2_ fold change of significantly upregulated or downregulated genes relative to young sham; fold-change ≥ 1.5, FDR ≤ 0.1. (Right) Percentage and total numbers of genes significantly upregulated (yellow) or downregulated (blue) relative to young sham. (**F**–**H**) Heatmaps of differentially regulated gene signatures showing extracellular matrix (ECM) genes (**F**), extracellular matrix remodeling genes (**G**), and prosurvival/proliferation genes (**H**), displayed as *Z* scores of RPKM.

We and others have demonstrated that, while bleomycin-induced lung fibrosis in young mice is self-resolving, aged mice exposed to bleomycin developed progressive lung fibrosis, which fails to resolve (14, 16). To identify transcriptional changes in aged lung fibroblasts that impede fibrosis resolution, we carried out bulk RNA-Seq on fibroblasts sorted by FACS (EpCAM^–^, CD45^–^, CD31^–^, GFP^+^), isolated from the lungs of young (2 months) and aged (18 months) Col1a1-GFP transgenic mice during the initial phase of lung fibrosis resolution (30 days after intratracheal bleomycin instillation) (Figure 1A). We compared the transcriptome of aged lung fibroblasts during persistent lung fibrosis with that of young lung fibroblasts during the resolution phase after bleomycin challenge. The transcriptome of young lung fibroblasts during fibrosis resolution was previously published (9) and served as a comparison in the current study.

Principal component analysis (PCA) revealed that lung fibroblast samples clustered in 4 different groups according to treatment (sham- and bleomycin-treated) and age (2 months versus 18 months). This analysis further revealed that, relative to injured fibroblasts from aged lungs, fibroblasts from injured young lungs were transcriptionally more similar to those from uninjured young lungs, suggesting that the magnitude of fibroblast activation at this time point following injury is more pronounced with aging (Figure 1B). To identify aging-associated alterations in the transcriptome of lung fibroblasts, we first compared the transcriptome of young lung fibroblasts with that of aged lung fibroblasts in the absence of injury. We identified 1200 differentially regulated genes, of which 69.7% were upregulated and 30.3% were downregulated (Figure 1C). Among the most highly expressed genes, ECM-related genes such as *Col1a1*, *Col1a2*, *Col3a1*, and *Sparc* were significantly downregulated, while inflammation-associated genes, including *Il6*, *Il1b*, and *Cxcl1*, were significantly upregulated in aged lung fibroblasts (Figure 1C). These data suggest that aged lung fibroblasts were more inflammatory and exhibited lower expression of ECM-related genes than young fibroblasts, and this is consistent with previous findings in mice and humans demonstrating a progressive increase of inflammation and reduced expression of ECM-related genes with aging (21, 22).

### Aging perpetuates profibrotic and prosurvival gene signatures in lung fibroblasts following bleomycin challenge.

We next compared the transcriptome of fibroblasts from injured young lungs with that of fibroblasts from uninjured young lungs. We identified 2421 differentially regulated genes in injured young lung fibroblasts, of which 62.8% were upregulated and 37.2% were downregulated (Figure 1D). Among the most upregulated and statistically significant genes were those previously implicated in fibroblast activation — including *Fn1* and *Spp1* (7, 23), encoding for fibronectin-1 and osteopontin, respectively (Figure 1D). Intriguingly, the myofibroblast gene markers *Acta2* and *Col1a1* were downregulated and unchanged, respectively, in young fibroblasts at this point of resolution after injury (Figure 1D), confirming our previous findings that fibroblasts from young mice reverse their state of activation during the resolution phase after bleomycin challenge compared with the peak of fibrosis (9).

We next compared the transcriptome of fibroblasts from injured aged lungs with that of fibroblasts from uninjured young lungs and identified 3596 differentially regulated genes in injured aged lung fibroblasts, of which 55.5% were upregulated and 44.5% were downregulated (Figure 1E). In contrast to fibroblasts from young lungs after injury, fibroblasts from aged lungs exhibited elevated expression of genes — including *Col1a1*, *Tnc*, *Timp1*, and *Cthrc1 —* associated with fibroblast activation and ECM remodeling (Figure 1E). These findings are consistent with our previous observations demonstrating persistent ECM protein accumulation in the lungs of aged mice following bleomycin challenge (16, 17).

To illustrate and directly compare the transcriptional changes in fibroblasts from young and aged lungs following injury, we generated heatmaps of selected genes that are representative of specific signaling pathways implicated in lung fibrosis, such as ECM remodeling and prosurvival pathways. As shown in Figure 1F, we found that the expression of genes encoding for ECM proteins was moderately increased in injured fibroblasts from young lungs compared with uninjured young lung fibroblasts during the early resolution phase of fibrosis. On the contrary, the expression of these genes remained strongly elevated in aged lung fibroblasts during the same time after bleomycin injury. Furthermore, the expression of ECM remodeling genes, including that of genes encoding for matrix-degrading enzymes, was elevated in young lung fibroblasts after injury, suggesting that ECM degradation may contribute to fibrosis resolution in young lungs. Aged fibroblasts, however, exhibited reduced expression of these genes compared with young ones, which may underpin the accumulation of collagen and the persistent fibrosis in aged lungs (Figure 1G). Furthermore, fibroblasts from injured aged lungs also exhibited marked elevation of prosurvival/proliferation genes, including *Birc5*, *Foxm1*, *Aurkb*, and *Plk1*, compared with those from injured young fibroblasts (Figure 1H). Interestingly, the transcription factor FOXM1 has been recently found to play a key role in lung fibrogenesis through the upregulation of profibrotic and prosurvival mediators, including BIRC5 and PLK1, in pathogenic lung fibroblasts (24).

Collectively, these data demonstrate that lung fibroblasts from aged mice after bleomycin injury exhibited altered gene expression signatures compared with those from young mice, including increased expression of genes encoding for ECM proteins and reduced expression of those implicated in ECM protein degradation and resorption. Additionally, numerous genes associated with inflammation and apoptosis resistance were markedly increased in aged lung fibroblasts compared with young fibroblasts after bleomycin challenge, suggesting that increased fibroblast activation/survival may be responsible for pathogenic fibroblast accumulation and increased collagen deposition in fibrotic aged lungs.

### PIM1 and NFATc1 are putative mediators of the sustained fibroblast activation in fibrotic aged lungs.

**Figure 2:**
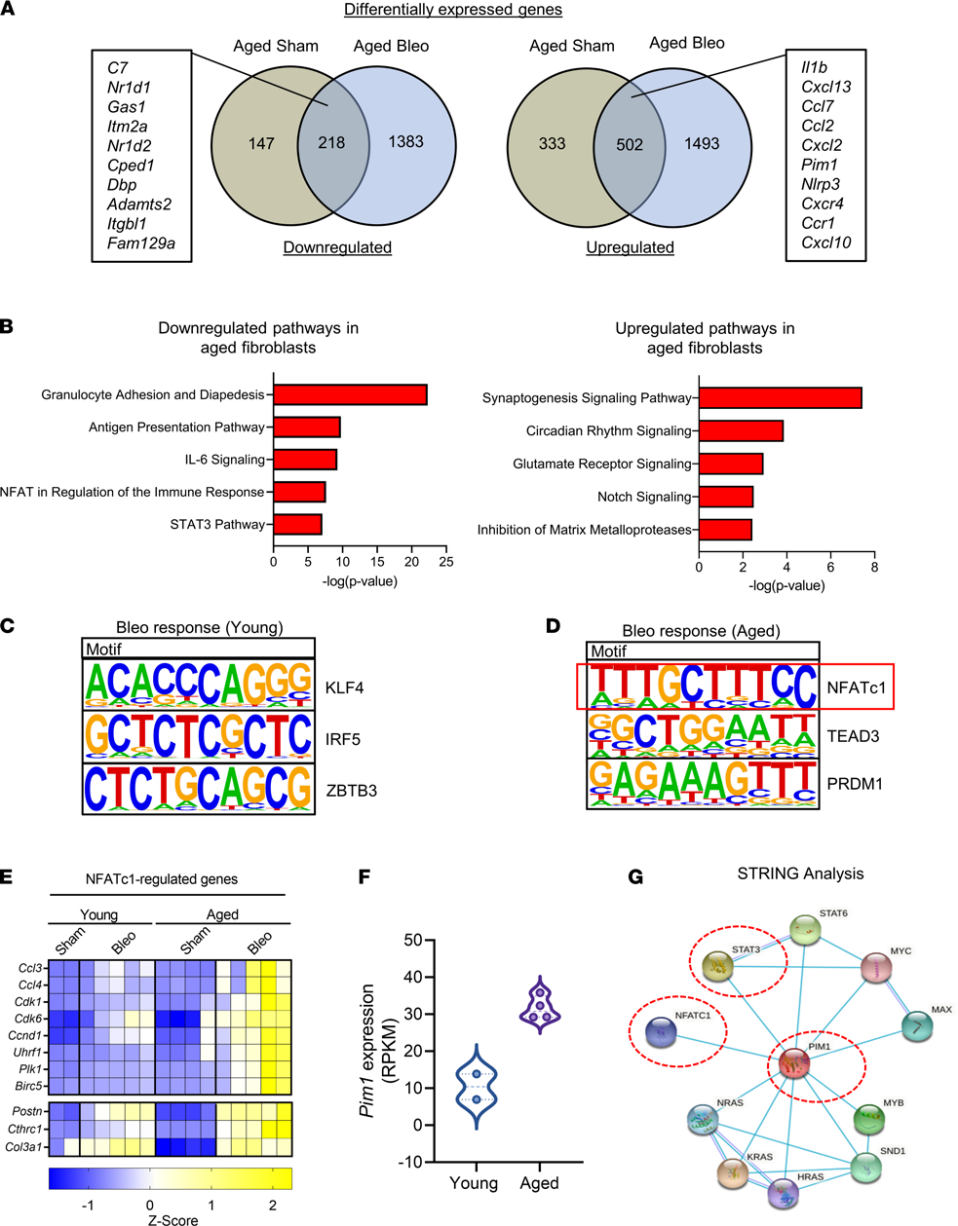
PIM1 and NFATc1 signaling pathways are associated with impaired fibrosis resolution in aged mice after bleomycin injury. (**A**) Venn diagrams of differentially regulated genes from uninjured aged and injured aged fibroblasts relative to uninjured young fibroblasts. Intersection genes with the highest average expression are displayed. (**B**) Ingenuity pathway analysis of the intersection genes identifies differentially regulated canonical pathways. (**C**) De novo transcription factor binding motif analysis of differentially expressed genes in young fibroblasts after injury showing the top 3 binding motif and their associated transcription factors. (**D**) De novo transcription factor binding motif analysis of differentially expressed genes in aged lung fibroblasts after injury showing the top 3 binding motif and their associated transcription factor. Red box indicates highest ranked transcription factor binding motif and associated transcription factor in aged lung fibroblasts. (**E**) Heatmap of genes with NFATc1 binding motifs displayed as *Z* scores of RPKM (young sham, *n* = 2; young bleo 30 days, *n* = 5; aged sham, *n* = 4; aged bleo 30 days, *n* = 5). (**F**) Expression of *PIM1* gene in young and aged lung fibroblasts (young sham, *n* = 2; aged sham, *n* = 4). (**G**) STRING functional protein association networks showing an interconnection between PIM1, STAT3, and NFATc1. Each node represents a protein, and each line represents a curated interaction.

To identify aging-associated genes that are responsible for the sustained fibrogenic and prosurvival gene signatures in aged lung fibroblasts after injury, we focused on genes whose expression was increased or reduced in aged lung fibroblasts compared with young ones (aging comparison) and maintained the same trend following bleomycin challenge (bleomycin response comparison). We identified a total of 720 differentially expressed genes (218 downregulated and 502 upregulated) that were shared between the aging comparison and the bleomycin response comparison (Figure 2A). Genes such as *Nr1d1*, *Adamts2,* and *Fam129a* were strongly downregulated in aged lung fibroblasts compared with young ones. Interestingly, it has been demonstrated that *Nr1d1*, encoding for Rev-Erbα, an important regulator of the circadian clock, regulates collagen homeostasis and that loss of its activity exacerbated bleomycin-induced pulmonary fibrosis (25, 26).

On the contrary, we found that numerous inflammatory-associated genes, including those encoding for cytokines and chemokine receptors, such as *Ccr1*, *Cxcr4*, *Ccl2*, *Il1b*, and *Cxcl10*, were upregulated in aged lung fibroblasts prior to and after bleomycin challenge (Figure 2A), suggesting that age-associated inflammation may influence fibroblast responses to injury and the recovery from fibrosis.

To identify signaling pathways that are involved in the transcriptional response of aged lung fibroblasts, we performed Ingenuity Pathway Analysis (IPA) and identified “Circadian Rhythm Signaling” pathway among the most downregulated pathways in aged lung fibroblasts (Figure 2B); this finding was consistent with the downregulation of 2 major regulators of this pathway, *Nr1d1* and *Nr1d2,* in aging. Among the upregulated pathways, multiple inflammatory pathways were enriched in aged lung fibroblasts, and among them were IL-6, NFAT, and STAT3 signaling pathways (Figure 2B).

Next, we sought to identify transcription factors that are responsible for the sustained profibrotic gene signature in aged lung fibroblasts after injury. To do that, we examined the promoter region (±2 kb from transcriptional start sites) of the differentially expressed genes in injured young and aged lung fibroblasts relative to uninjured fibroblasts using HOMER (27). We found that, in injured young lung fibroblasts, a large portion of differentially expressed genes contained a Krüppel-like factor 4 (KLF4) binding motif (Figure 2C). Interestingly, an antifibrotic function for KLF4 has been previously identified in lung and kidney fibrosis (28–30), suggesting that this transcription factor may be implicated in attenuating lung profibrotic responses in young mice after bleomycin challenge.

Next, we found that approximately a third of differentially expressed genes in injured aged lung fibroblasts contained the binding motif for NFATc1 (Figure 2D). Intriguingly, the NFAT signaling pathway was among the top enriched pathways in aged lung fibroblasts following injury, as demonstrated by our IPA analysis (Figure 2B). Genes containing NFATc1 binding sites that exhibited increased expression in aged lung fibroblasts include those encoding for chemokines such as *Ccl3* and *Ccl4*; proliferation/prosurvival proteins, such as *Ccnd1*, *Cdk1*, *Plk1*, and *Birc5*; as well as ECM proteins including *Postn, Col3a1,* and *Cthrc1* (Figure 2E). Several of these genes have been demonstrated to be direct targets of NFATc1 (31, 32), increasing confidence in our findings.

Interestingly, the expression of *Postn*, *Cthrc1*, and *Col3a1* was strongly enriched in a pathogenic fibroblast population that was recently discovered in fibrotic mouse lungs, as well as in IPF lungs (7, 33). Of note, all 3 genes contain NFATc1 binding motifs in their regulatory regions, suggesting that NFATc1 may perpetuate aberrant transcriptional programs in this fibroblast population. NFATc1 is a transcription factor implicated in T cell biology and innate immune responses (34). Although NFATc1 is expressed in mesenchymal cells (35), its role in fibroblast activation and lung fibrosis is largely unknown. Multiple receptors, including GPCRs, as well protein kinases such as protein kinase A and PIM1, rely on NFATc1 activity to achieve critical cellular functions including proliferation and apoptosis (36, 37). Intriguingly, the *PIM1* gene, encoding for the kinase PIM1, was one of the top upregulated genes in lung fibroblasts from aged mice (Figure 2F), and its function was previously implicated in STAT3-mediated cell survival and cancer-associated fibroblast activation (38, 39). Intriguingly, STRING analysis (40) confirmed the interconnection between PIM1, NFATc1, and STAT3, as shown in Figure 2G. These findings support the hypothesis that increased expression of PIM1 in aging leads to increased NFATc1 transcriptional activity, thereby perpetuating profibrotic gene signatures in aged lung fibroblasts after injury.

### The expression of PIM1 and NFATc1 is enriched in pathogenic fibroblasts in IPF lungs.

**Figure 3:**
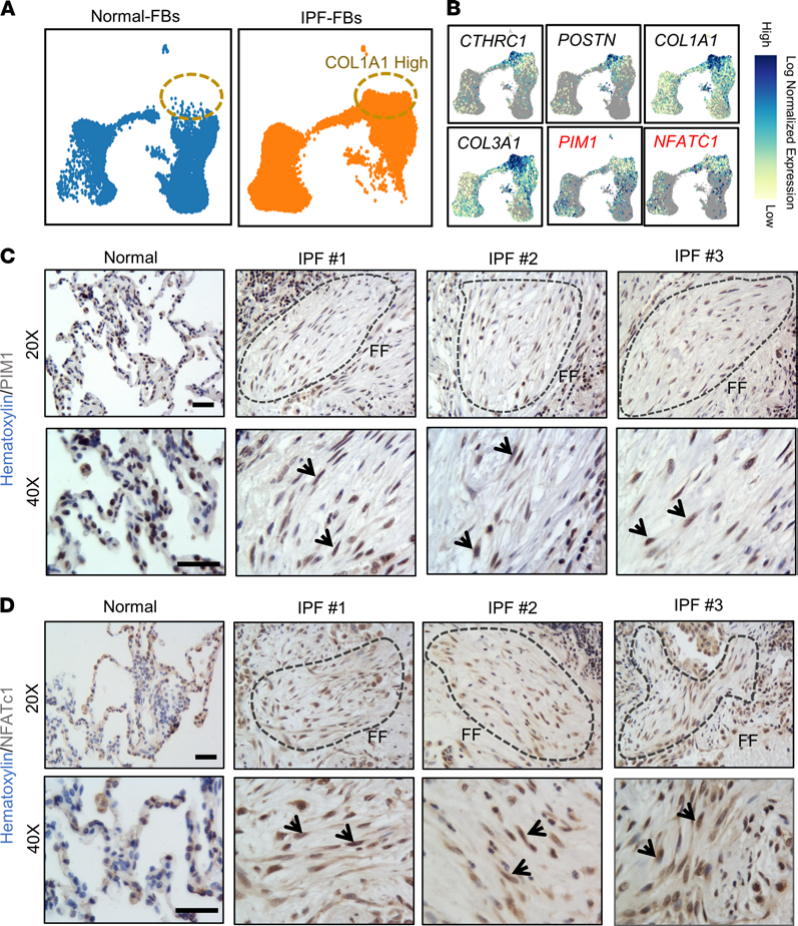
NFATc1 and PIM1 expression is enriched in pathogenic fibroblasts in IPF. (**A**) UMAP plots derived from publicly available scRNA-Seq data set (GSE132771) of mesenchymal cells sorted by FACS (CD31^–^CD45^–^CD235a^–^EpCAM^–^) isolated from normal and IPF lungs. (**B**) Normalized expression of *CTHRC1*, *POSTN*, *COL1A1*, *COL3A1*, *PIM1*, and *NFATc1* in normal and pathogenic lung fibroblast populations. (**C**) IHC of human normal and IPF lung sections showing PIM1 nuclear expression. Dotted lines indicate fibrotic foci (FF). Arrows show PIM1-positive nuclei. Scale bar: 50 μm. (**D**) IHC of human normal and IPF lung sections showing NFATc1 nuclear expression. Dotted lines indicate fibrotic foci (FF). Arrows show NFATc1-positive nuclei. Scale bar: 50 μm.

To investigate the relevance of the PIM1 signaling pathway in human IPF, we interrogated a publicly available scRNA-Seq data set from normal and IPF lungs (GSE132771) (7). We identified mesenchymal cells, epithelial cells, endothelial cells, and immune cells based on the expression of specific gene markers, and we reclustered the mesenchymal cell population using uniform manifold approximation and projection (UMAP). Similarly to what we found in fibroblasts from diseased aged mouse lungs, UMAP — along with gene expression analysis — revealed increased expression of genes associated with fibroblast activation, including *CTHRC1*, *POSTN*, *COL1A1*, and *COL3A1,* in a pathogenic fibroblast population in IPF lungs, as previously demonstrated (7) (Figure 3, A and B). Interestingly, *PIM1* and *NFATc1* expression was enriched in this pathogenic population, supporting a role for these factors in myofibroblast differentiation in human IPF lungs (Figure 3B). To complement our scRNA-Seq analysis and validate the expression of PIM1 and NFATc1 at the protein level in diseased human lungs, we performed an IHC analysis on normal and IPF lung tissues. While we observed widespread positivity for both NFATc1 and PIM1 in diseased lungs, consistent with their expression in a variety of cell types (41–44), we also confirmed their strong nuclear expression in fibroblastic foci (Figure 3, C and D), which represent areas of active fibrosis in human IPF. Collectively, our data suggest that PIM1 and NFATc1 may play direct roles in fibroblast activation and expansion in fibrotic human lungs.

### PIM1 potentiates TGF-β signaling in human lung fibroblasts, and this effect is attenuated by NFATc1 inhibition.

**Figure 4:**
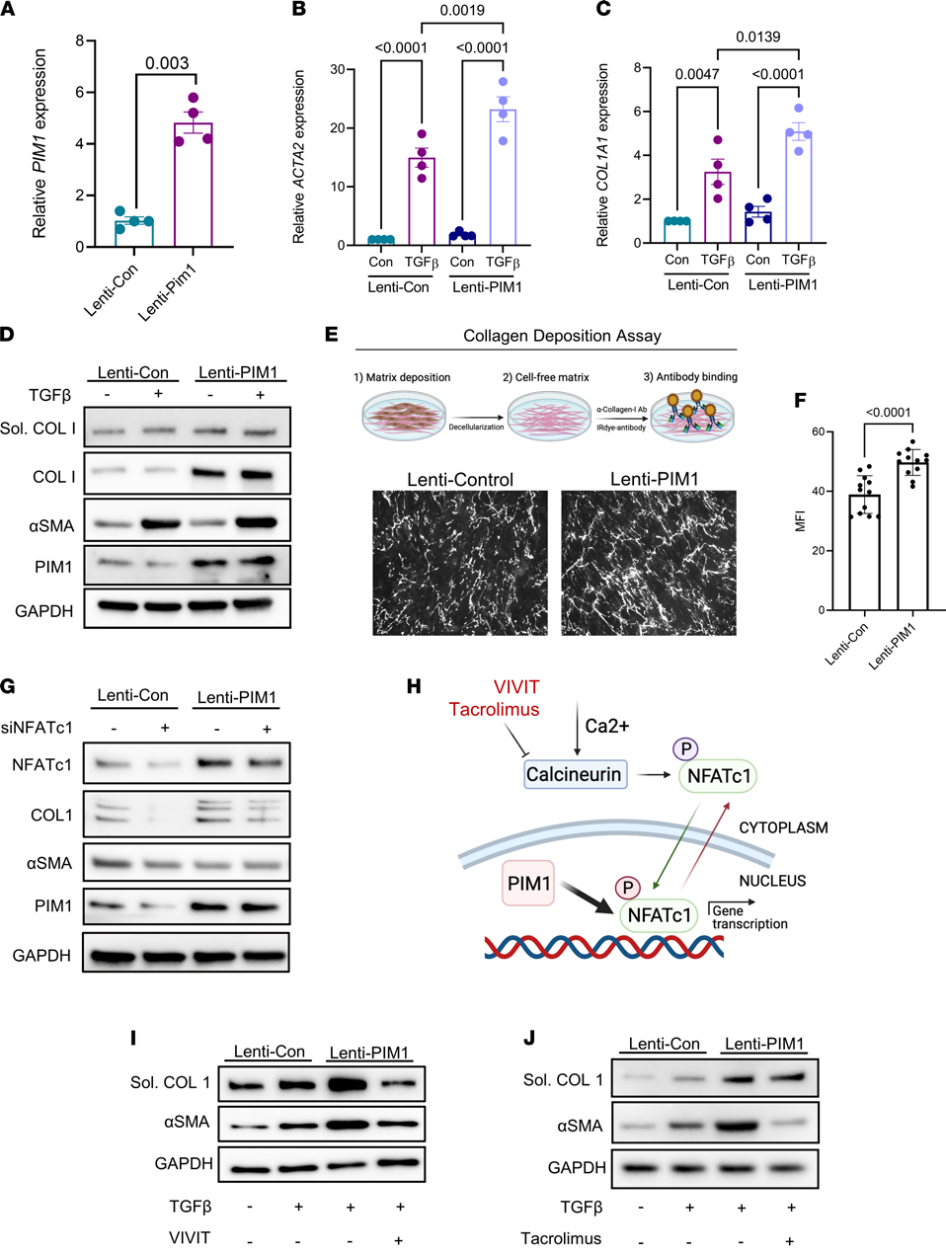
NFATc1 inhibition attenuates PIM1-promoted lung fibroblast activation. (**A**) Normal human lung fibroblasts transduced with control lentivirus (pLV-EGFP-CMV-mCherry) or lentivirus carrying the human *PIM1* gene (pLV-EGFP-CMV-hPIM1) for 48 hours, followed by FACS to collect GFP^+^ cells. Shown is the mRNA expression of PIM1 evaluated by qPCR. Data are shown as mean ± SEM of *n* = 4 independent experiments. *P* values were calculated using 2-tailed, paired Student’s *t* test. (**B** and **C**) mRNA expression of *ACTA2* (**B**) and *COL1A1* (**C**) in control and PIM1 overexpressing cells treated with 2 ng/mL of TGF-β for 24 hours. Data are shown as mean ± SEM of *n* = 4 independent experiments. *P* values were calculated using 1-way ANOVA with Holm-Šidák post hoc test. (**D**) Normal human lung fibroblasts transduced with control lentivirus or PIM1 lentivirus were treated with 2 ng/mL of TGF-β for 24 and then analyzed by Western blotting. Shown is a representative blot of gels run in parallel. (**E**) Representative images of collagen-I fibers from a collagen deposition assay carried out in control and PIM1-overexpressing cells. (**F**) Quantification of collagen-I secretion measured as mean fluorescence intensity. *n* = 12. Data are shown as mean ± SEM. *P* values were calculated using 2-tailed Student’s *t* test. (**G**) Control and PIM1-overexpressing cells were transfected with scrambled or NFATc1 siRNAs for 48 hours, followed by Western blotting analysis. Shown is a representative blot of 3 independent experiments. (**H**) Schematic showing mechanisms of NFATc1 inhibition by VIVIT and tacrolimus. Following phosphorylation, NFATc1 is sequestered in the cytoplasm. Upon dephosphorylation by calcineurin, NFATc1 translocates into the nucleus, where it becomes transcriptionally active. In the nucleus, PIM1 can phosphorylate NFATc1 to enhance its transcription. (**I** and **J**) Western blotting analysis of control and PIM1 overexpressing lung fibroblasts cotreated with 2 ng/mL of TGF-β and the NFATc1 inhibitors VIVIT (5 μM) and tacrolimus (1 μM). Shown is a representative blot of 3 independent experiments.

To translate these findings to human cells and evaluate the fibrogenic effect of PIM1 in lung fibroblast activation in vitro, we first overexpressed this protein kinase in normal human lung fibroblasts using lentiviral transduction. Compared with lung fibroblasts transduced with control lentivirus, PIM1-overexpressing lung fibroblasts exhibited about a 4-fold increase in *PIM1* gene expression, which was comparable with that observed in aged mouse lung fibroblasts (Figure 4A). To investigate the contribution of PIM1 to TGF-β–induced lung fibroblast activation, we exposed control and PIM1-overexpressing cells to TGF-β for 24 hours, followed by quantitative PCR (qPCR) analysis to evaluate expression of profibrotic genes such as *ACTA2* and *COL1A1*. As shown in Figure 4, B and C, overexpression of PIM1 did not increase basal level expression of *ACTA2* and *COL1A1* genes; however, it potentiated TGF-β–induced expression of these profibrotic genes. At a protein level, overexpression of PIM1 recapitulated what we observed at an RNA level except for collagen-I, whose protein expression, as well as secretion, was increased in PIM1-overexpressing cells even in the absence of TGF-β (Figure 4D). This latter observation was also confirmed using a collagen deposition assay (Figure 4, E and F) (16), further demonstrating that PIM1 is directly involved in fibroblast collagen synthesis and/or secretion.

To investigate whether the increased collagen protein expression induced by PIM1 overexpression was attenuated by NFATc1 inhibition, we silenced this transcription factor in PIM1-overexpressing cells using RNA interference (RNAi). As shown in Figure 4G, PIM1 overexpression increased collagen levels and this effect was attenuated by NFATc1 knockdown. Of note, PIM1 overexpression upregulated NFATc1 protein levels, and NFATc1 silencing inhibited PIM1 expression in control cells, suggesting a positive feedback loop between NFATc1 and PIM1 in support of fibroblast activation.

Several inhibitors of NFATc1 have been developed to block the translocation of this transcription factor into the nucleus (45, 46). To further validate the interconnection between NFATc1 and PIM1, we pharmacologically inhibited NFATc1 signaling pathways using VIVIT and tacrolimus. VIVIT is a cell-permeable peptide that inhibits the nuclear translocation of NFATc1 (45), and tacrolimus is a macrolide lactone that inhibits calcineurin-dependent dephosphorylation of NFATc1, which is required for its nuclear translocation (46) (Figure 4H). PIM1-overexpressing lung fibroblasts were treated with TGF-β in the presence or absence of VIVIT or tacrolimus. In PIM1-overexpressing cells, TGF-β elevated the protein levels of α-SMA to a higher level than in control cells; however, this effect was fully abrogated by both VIVIT and tacrolimus (Figure 4, I and J). Similarly, PIM1-promoted collagen-I secretion was also blocked by VIVIT and, to a lesser extent, by tacrolimus. Our data demonstrate that overexpression of PIM1 in normal lung fibroblasts increases collagen synthesis and potentiates TGF-β signaling, and this effect is attenuated by NFATc1 inhibition.

### Inhibition of PIM1 and NFATc1 attenuates IPF-derived lung fibroblast activation.

**Figure 5:**
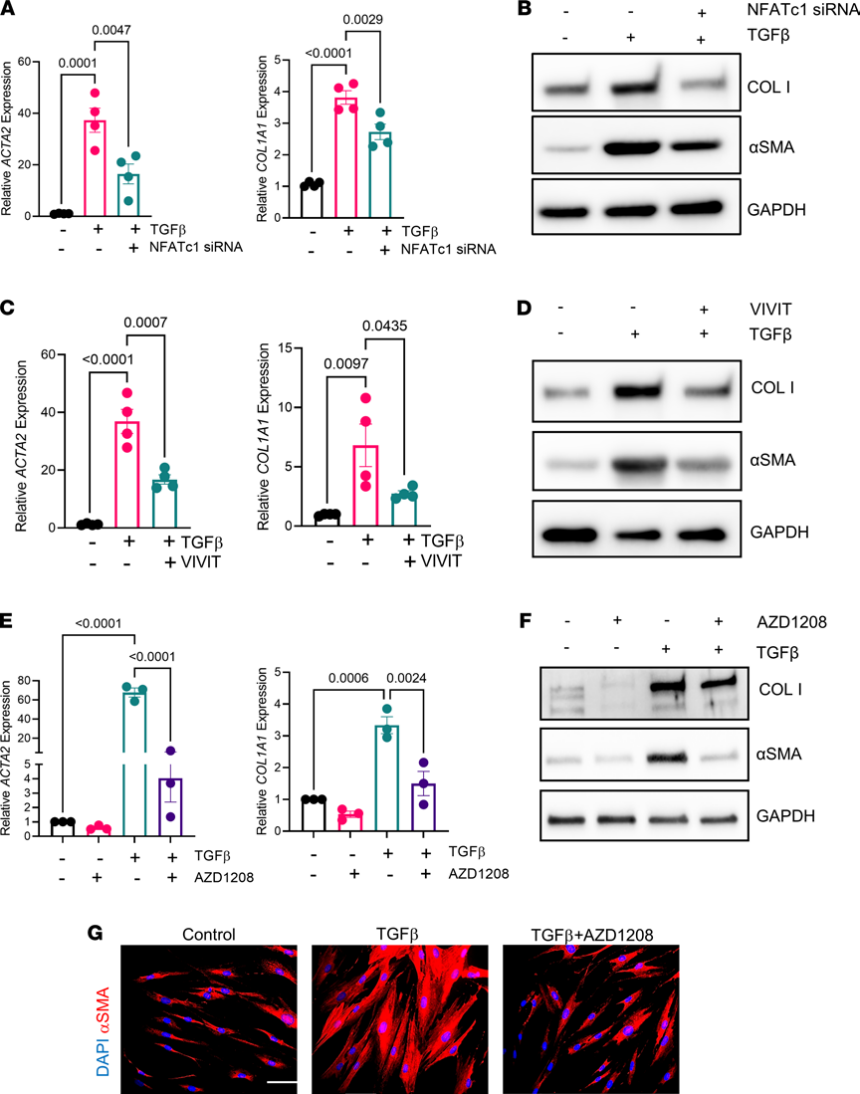
Inhibition of PIM1 signaling pathway reduces profibrotic gene expression in IPF-derived lung fibroblasts. (**A**) qPCR analysis of profibrotic gene expression in IPF-derived lung fibroblasts transfected with scrambled siRNA or siRNA for NFATc1 for 48 hours, followed by treatment with 2 ng/mL of TGF-β for an additional 24 hours. Data are shown as mean ± SEM of *n* = 4 independent experiments. *P* values were calculated using 1-way ANOVA with Holm-Šidák post hoc test. (**B**) IPF-derived lung fibroblasts were transfected with scrambled or NFATc1 siRNAs for 48 hours and then treated with 2 ng/mL of TGF-β for an additional 24 hours, followed by Western blotting analysis. Shown is a representative blot of 3 independent experiments. (**C**) qPCR analysis of profibrotic gene expression in IPF-derived lung fibroblasts treated with VIVIT (5 μM) and 2 ng/mL of TGF-β for 24 hours. Data are shown as mean ± SEM of *n* = 4 independent experiments. *P* values were calculated using 1-way ANOVA with Holm-Šidák post hoc test. (**D**) IPF-derived lung fibroblasts were treated with VIVIT (5 μM) and 2 ng/mL of TGF-β for 24 hours and analyzed via Western blot. Shown is a representative blot of *n* = 2 independent experiments. (**E**) qPCR analysis of profibrotic genes in IPF-derived lung fibroblasts cotreated with 10 μM of AZD1208 and 2 ng/mL of TGF-β for 24 hours. Data are shown as mean ± SEM of *n* = 3 independent experiments. *P* values were calculated using 1-way ANOVA with Holm-Šidák post hoc test. (**F**) IPF-derived lung fibroblasts were cotreated with 10 μM of AZD1208 and 2 ng/mL of TGF-β for 24 hours and analyzed by Western blotting. Shown is a representative blot of 3 independent experiments. (**G**) IHC of IPF-derived fibroblasts cotreated with 10 μM of AZD1208 and 2 ng/mL of TGF-β for 24 hours. Scale bar: 50 μm. Representative images of 3 independent experiments are shown.

To investigate the individual contribution of NFATc1 and PIM1 to IPF-derived lung fibroblast activation, we first performed RNAi-mediated knockdown of NFATc1 in primary lung fibroblasts derived from 3 independent IPF patients, followed by treatment with TGF-β to enhance their activation. As shown in Figure 5A, knockdown of NFATc1 significantly attenuated TGF-β–induced expression of *ACTA2* and *COL1A1*, and this effect was mirrored at the protein level (Figure 5B). Similarly, inhibition of NFATc1 by VIVIT strongly inhibited TGF-β–induced expression of *ACTA2* and *COL1A1* at the mRNA, as well as protein levels (Figure 5, C and D).

Next, we pharmacologically targeted PIM1 kinase in IPF-derived lung fibroblasts using AZD1208, a small molecule inhibitor that was developed for treatment of cancer patients (47). IPF-derived fibroblasts were treated with this small molecule in the presence or absence of TGF-β, followed by qPCR and Western blotting analysis. As shown in Figure 5, E and F, inhibition of PIM1 kinase attenuated both α-SMA and collagen-I expression in the absence of TGF-β, and a similar effect was also obtained when fibroblasts were exposed to TGF-β. Finally, immunofluorescence analysis of IPF-derived fibroblasts treated with AZD1208 demonstrated that inhibition of PIM1 blocked TGF-β–induced α-SMA fiber formation (Figure 5G). These findings demonstrate that both NFATc1 and PIM1 are profibrotic mediators and their inhibition can suppress the activation of IPF-derived lung fibroblasts.

### The PIM1/NFATc1 axis regulates aging-associated prosurvival genes, and pharmacologic inhibition of PIM1 sensitizes IPF-derived lung fibroblasts to apoptosis.

In our transcriptome analysis, we found that numerous prosurvival/proliferation-associated genes and transcriptional targets of NFATc1 — including *Foxm1*, *Birc5,* and *Plk1* — were upregulated in injured aged lung fibroblasts. Notably, both FOXM1 and BIRC5 were found elevated in IPF-derived lung fibroblasts (24), and conditional deletion of *Foxm1* in mouse fibroblasts accelerated lung fibrosis resolution following bleomycin challenge (24). These findings suggest that increased NFATc1 transcriptional activity may perpetuate survival and fibrogenic signals in aged lung fibroblasts, thereby impairing fibrosis resolution following injury.

**Figure 6:**
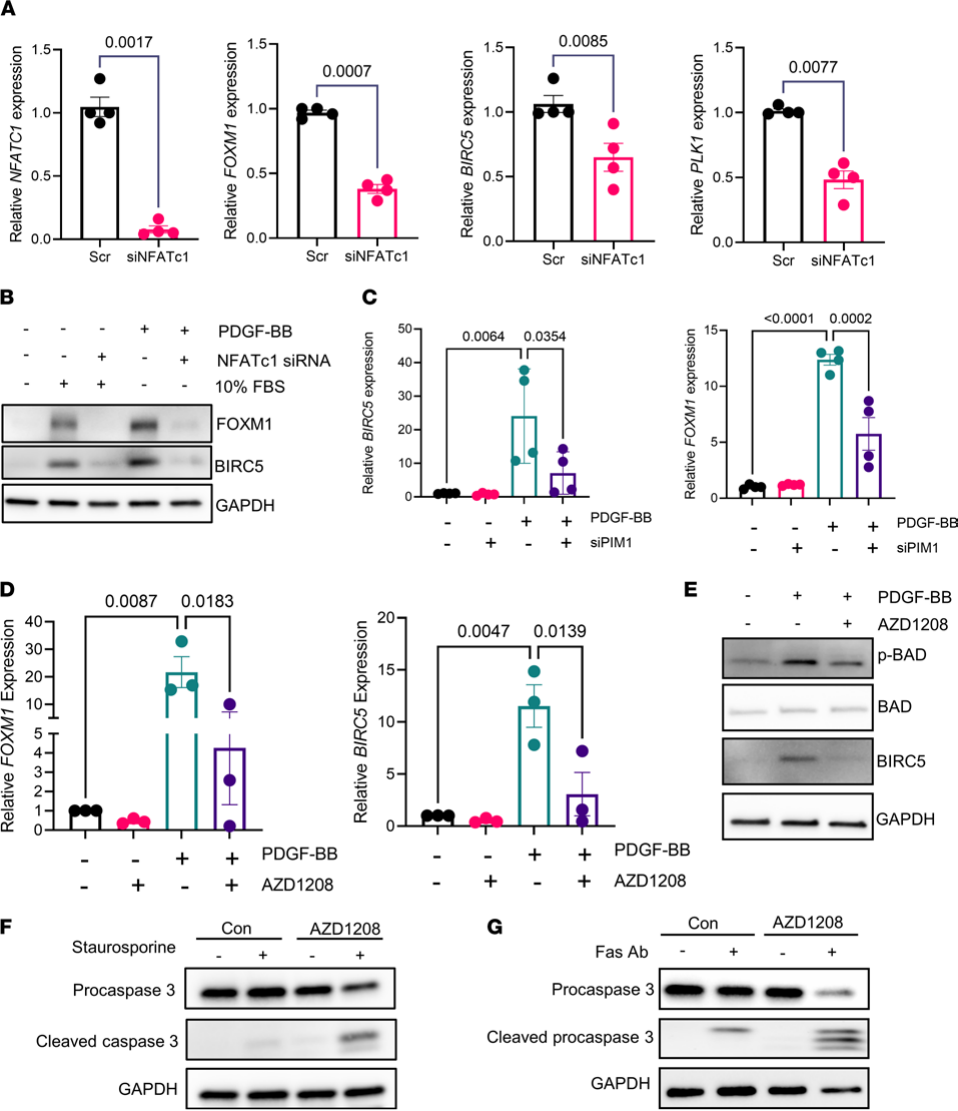
Inhibition of PIM1 signaling pathway reduces prosurvival gene expression and sensitizes IPF-derived lung fibroblasts to apoptotic cues. (**A**) qPCR analysis of aging-associated prosurvival genes and those with NFATc1 binding sites in IPF-derived fibroblasts transfected with scrambled siRNA or siRNA for NFATc1 for 48 hours. Data are shown as mean ± SEM of *n* = 4 independent experiments. *P* values were calculated using 2-tailed, paired Student’s *t* test. (**B**) Western blot analysis of FOXM1 and BIRC5 in IPF-derived fibroblasts transfected with scrambled siRNA or siNFATc1 for 48 hours and then stimulated with 50 ng/mL of PDGF-BB or 10% FBS for an additional 24 hours. Shown is a representative blot of 2 independent experiments. (**C**) qPCR analysis of prosurvival genes in IPF-derived fibroblasts transfected with scrambled siRNA or siRNA for PIM1 for 48 hours. Data are shown as mean ± SEM of *n* = 4 independent experiments. *P* values were calculated using 1-way ANOVA with Holm-Šidák post hoc test. (**D**) qPCR analysis of prosurvival gene expression in IPF-derived lung fibroblasts cotreated with 10 μM of AZD1208 and 50 ng/mL of PDGF-BB for 24 hours. Data are shown as mean ± SEM of *n* = 3 independent experiments. *P* values were calculated using 1-way ANOVA with Holm-Šidák post hoc test. (**E**) IPF-derived lung fibroblasts were cotreated with 10 μM of AZD1208 and 50 ng/mL of PDGF-BB for 24 hours and analyzed by Western blotting using antibodies against phospho-BAD, total-BAD, BIRC5, and GAPDH. (**F**) IPF-derived lung fibroblasts were pretreated for 24 hours with 10 μM of AZD1208 and with 300 nM of staurosporine for an additional 2 hours, followed by Western blotting analysis using antibodies against procaspase-3, cleaved caspase-3, and GAPDH. (**G**) IPF-derived lung fibroblasts were preincubated for 24 hours with 10 μM of AZD1208, followed by treatment with 0.5 μg/mL of FAS-activating antibody for an additional 24 hours. Procaspase-3 and cleaved caspase-3 were detected by Western blotting. Shown is a representative blot of 3 independent experiments.

To directly evaluate the contribution of NFATc1 to the expression of aging-associated prosurvival genes, we silenced NFATc1 in IPF fibroblasts using RNAi and demonstrated significantly reduced expression of *FOXM1*, *BIRC5*, and *PLK1* genes in these cells relative to control lung fibroblasts (Figure 6A). We also found that serum and PDGF-BB, both implicated in fibroblast activation in vitro (48, 49), potently stimulated FOXM1 and BIRC5 expression in IPF fibroblasts, and this effect was abrogated by NFATc1 silencing (Figure 6B). Similarly, inhibition of PIM1 using RNAi or AZD1208 also attenuated the expression of *BIRC5* and *FOXM1* induced by PDGF-BB (Figure 6, C and D), further demonstrating that PIM1 and NFATc1 share common downstream targets and fibrogenic mediators.

We also found that, in addition to attenuating PDGF-BB–induced prosurvival gene transcription, AZD1208 inhibited the phosphorylation of Bcl2-associated agonist of cell death (BAD) (Figure 6E), a proapoptotic protein that is inhibited by phosphorylation and has been demonstrated to be a direct target of PIM1 (50, 51). As expected, PIM1-overexpressing lung fibroblasts exhibited increased BAD phosphorylation and were resistant to FAS activating antibody–induced caspase-3 activation, a key effector of apoptosis (Supplemental Figure 1, A and B; supplemental material available online with this article; https://doi.org/10.1172/jci.insight.153672DS1). Since it has been shown that IPF is characterized by the accumulation of apoptosis-resistant myofibroblasts (52–54), we also evaluated whether inhibition of PIM1 by AZD1208 can sensitize IPF-derived fibroblasts to apoptosis. As shown in Figure 6F, PIM1 inhibition in IPF fibroblasts potentiated staurosporine-promoted caspase-3 cleavage. Similarly, PIM1 inhibition also potentiated caspase-3 cleavage induced by FAS-activating antibody (Figure 6G). Interestingly, resistance from FAS-induced apoptosis was recently implicated as a mechanism that contributed to persistent lung fibrosis in aged mice (14), and inhibition of this apoptotic pathway in fibroblasts contributed to their pathogenic activation and persistence in the lung after injury (11).

To assess the direct contribution of NFATc1 to apoptosis resistance, we inhibited NFATc1 in PIM1-overexpressing or IPF-derived lung fibroblasts using siRNA or VIVIT/tacrolimus in the presence of a FAS-activating antibody. As shown in Supplemental Figure 2, A and B, inhibition of NFATc1 alone in PIM1-overexpressing or IPF-derived lung fibroblasts did not affect FAS antibody–induced caspase-3 cleavage. These data demonstrate that, while NFATc1 inhibition in IPF-derived lung fibroblasts attenuated the transcription of prosurvival genes, this effect was not sufficient to promote or enhance caspase cleavage by FAS signaling activation. These data suggest that FAS antibody–promoted apoptosis in these cells do not rely on de novo transcription of prosurvival genes by NFATc1 but rather on posttranslational mechanisms.

Collectively, our data demonstrate that inhibition of PIM1 signaling pathway sensitized IPF-derived lung fibroblasts to apoptosis, suggesting that targeting this prosurvival pathway may be an effective strategy to promote myofibroblast apoptosis and fibrosis resolution in IPF.

### Pharmacological inhibition of PIM1 signaling pathway attenuates profibrotic gene expression and collagen secretion in human IPF organotypic lung cultures ex vivo.

**Figure 7:**
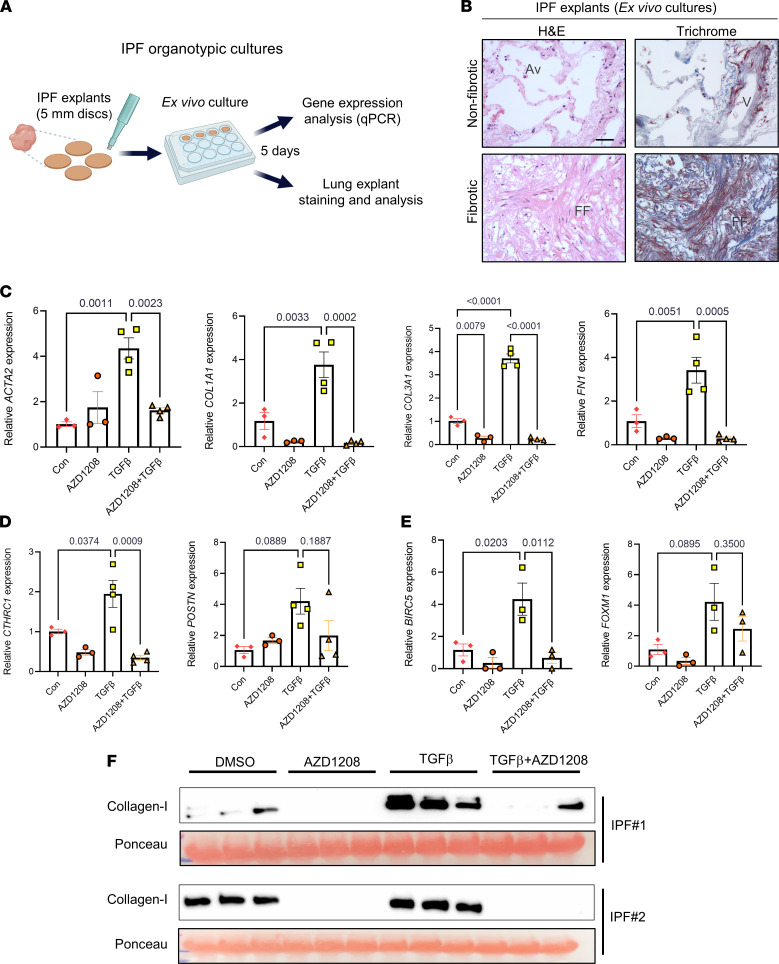
PIM1 inhibition inhibits profibrotic gene expression and collagen secretion in organotypic IPF lung cultures ex vivo. (**A**) Schematic of organotypic culture of IPF lungs. (**B**) H&E and trichrome staining of IPF lung tissue explants after 5 days in culture showing intact tissue architecture of nonfibrotic area (top panels) and distorted architecture of fibrotic area (bottom panels). Scale bar: 50 μm. FF, fibrotic foci; V, vein; Av, alveoli. (**C**) qPCR analysis of ECM gene expression in IPF lung explants treated with 10 μM of AZD1208 or DMSO control in combination with or without 10 ng/mL of TGF-β for 5 days. *n* ≥ 3 IPF lung explants. Data are shown as mean ± SEM. *P* values were calculated using 1-way ANOVA with Holm-Šidák post hoc test. (**D**) qPCR analysis of pathogenic lung fibroblasts gene markers in IPF lung explants treated with 10 μM of AZD1208 or DMSO in the presence or absence of 10 ng/mL of TGF-β for 5 days. *n* ≥ 3 IPF lung explants. Data are shown as mean ± SEM. *P* values were calculated using 1-way ANOVA with Holm-Šidák post hoc test. (**E**) qPCR analysis of prosurvival genes in IPF lung explants treated with 10 μM of AZD1208 or DMSO in the presence or absence of 10 ng/mL of TGF-β for 5 days. *n* = 3 IPF lung explants. Data are shown as mean ± SEM. *P* values were calculated using 1-way ANOVA with Holm-Šidák post hoc test. (**F**) Soluble collagen-I secreted from IPF lung explants into the media was evaluated by Western blot analysis. Each lane contained equal volume of conditioned medium of different lung sections obtained from single IPF lung explants. Ponceau staining was used as loading control for secreted collagen-I.

To explore the therapeutic potential of interrupting the PIM1 signaling pathway in a more relevant disease setting, we developed organotypic cultures from human IPF lungs ex vivo and tested the capacity of PIM1 and NFATc1 inhibitors to attenuate profibrotic gene expression in these explants in the presence or absence of TGF-β (Figure 7A). Lung explants (IPF discs) were generated using a biopsy puncher and cultured for 5 days in the presence or absence of inhibitors. To evaluate whether lung architecture was preserved following ex vivo culture, we carried out H&E and trichrome staining of the lung explants and demonstrated that nonfibrotic areas of the lungs maintained a relatively intact alveolar structure, while fibrotic areas exhibited fibroblastic foci and abundant collagen deposition (Figure 7B). In addition, live/dead staining, together with FACS analysis, demonstrated minimal cell mortality in these lung explants after several days of culture (data not shown). To first assess whether inhibiting NFATc1-mediated transcription restricted fibroblast activation and limited collagen secretion in IPF lung cultures ex vivo, we treated these diseased lung explants with VIVIT or tacrolimus in the presence or absence of TGF-β for 5 days, followed by qPCR and Western blotting analysis to measure profibrotic gene expression and collagen accumulation, respectively (Supplemental Figure 3, A and B). qPCR analysis showed that TGF-β consistently upregulated profibrotic gene expression in these lung explants; however, this effect was only partially blocked by NFATc1 inhibitors. In fact, while tacrolimus showed no beneficial effects on TGF-β–induced profibrotic gene expression, NFATc1 inhibition by VIVIT led to reduced expression of a subset of genes associated with myofibroblast differentiation, including *ACTA2* and *POSTN*. Western blotting analysis of secreted collagen-I released in the culture media by the IPF explants confirmed the RNA data showing either no change or increased collagen secretion by tacrolimus. Inhibition of NFATc1 by VIVIT, however, attenuated collagen secretion in 2 of 3 explants, suggesting that a more selective strategy for NFATc1 inhibition may be necessary to fully achieve myofibroblast deactivation in a complex disease setting.

Next, to further investigate whether inhibiting PIM1 in IPF lung explants led to reduced fibroblast activation and collagen secretion, we treated IPF lung explants with AZD1208 in the presence or absence of TGF-β for 5 days. qPCR expression analysis demonstrated that IPF explants treated with PIM1 inhibitor markedly reduced basal expression of ECM genes and those induced by TGF-β (Figure 7C). Furthermore, the expression of the pathogenic lung fibroblast marker, *CTHRC1,* and the prosurvival gene, *BIRC5*, were significantly reduced by PIM1 inhibition in these diseased lung explants (Figure 7, D and E). To examine whether inhibition of *COL1A1* gene transcription by AZD1208 led to diminished protein secretion, we measured the amount of secreted collagen-I using Western blotting. As shown in Figure 7F, inhibition of PIM1 in IPF lung explants strongly abrogated basal and TGF-β–induced collagen secretion, confirming our transcriptional analysis. Taken together, these data implicate the PIM1 signaling pathway in the sustained activation of profibrotic signals in IPF lungs ex vivo, suggesting that inhibition of this pathway in IPF patients may be an efficacious strategy to promote lung fibroblast quiescence and limit disease progression.

## Discussion

Progressive organ fibrosis is the result of a dysfunctional wound healing response in which activated fibroblasts (myofibroblasts) aberrantly produce and secrete ECM proteins, leading to lung scarring (1). As seen in other fibrotic tissues, lungs from IPF patients exhibit abnormal myofibroblast accumulation and excessive collagen deposition, resulting in distortion of lung architecture and loss of organ functions (55).

By applying the bleomycin model of lung fibrosis to aged mice we have recently demonstrated that fibrosis in these animals is persistent following a single dose of bleomycin, and similarly to IPF lungs, injured aged mouse lungs were characterized by the progressive accumulation of myofibroblasts and by the abundant deposition of collagen (16, 17). Other groups have also characterized the persistent nature of fibrosis in aged mice following a single dose of bleomycin and have demonstrated that aging altered multiple signaling pathways in lung fibroblasts, including those related to metabolism and oxidative stress (13–15).

By taking advantage of the self-resolving nature of lung fibrosis in young mice following a single dose of bleomycin, we recently showed that the expression of profibrotic and prosurvival genes were strongly elevated during the peak of fibrosis (day 14), followed by a return to baseline during the initial resolution phase (day 30), suggesting the existence of transcriptional programs in young mice that restore fibroblast quiescence following lung injury (9).

Lineage tracing studies have shown that, following bleomycin injury, lung lipofibroblasts differentiated into myofibroblasts, and this process was followed by the spontaneous reversion of these scar-forming cells to quiescent lipofibroblasts during fibrosis resolution (56). In line with this evidence, we recently demonstrated that myofibroblast dedifferentiation proceeded through distinct transcriptional programs and that dedifferentiation may facilitate their removal through apoptosis (57).

Here, we discovered that, during the early resolution phase after bleomycin-induced lung fibrosis, aged lung fibroblasts failed to return to a quiescent state, as demonstrated by the elevated expression of profibrotic and prosurvival genes, which largely overlapped with those enriched in young fibroblasts at the peak of fibrosis (day 14) (9). These initial findings led to the hypothesis that aging may perpetuate fibrogenic gene signatures that are typically associated with the acute fibrotic phase after bleomycin injury. In fact, our transcriptional analysis demonstrated that injured aged lung fibroblasts exhibited elevated expression of genes associated with inflammation, ECM remodeling, cell proliferation, and survival.

IPA identified several pathways and upstream regulators associated with inflammation that were enriched in aged lung fibroblasts, including IL-6, STAT3, and NFAT. These findings are consistent with previous reports demonstrating elevated low-grade inflammation, also known as “sterile inflammation,” in both mouse and human tissues (58). Intriguingly, the elevated expression of numerous inflammatory-associated genes was sustained in injured aged lung fibroblasts after bleomycin injury, suggesting that increased sterile inflammation in aging may perpetuate lung fibroblast activation and/or impair their return to quiescence. In this regard, we found that the expression of PIM1 was significantly elevated in aged lung fibroblasts, and its overexpression in normal lung fibroblasts in vitro enhanced their fibrogenic activation and promoted apoptosis resistance. This is consistent with previous reports from the cancer field demonstrating that PIM1 inhibited apoptosis in tumor cells (38) and enhanced ECM secretion in cancer-associated fibroblasts (39).

Among the inflammatory genes whose expression was elevated in aged lung fibroblasts were those encoding for chemoattractant factors, suggesting that immune cell recruitment to the injured aged lungs may contribute to the perpetuation of fibroblast activation. In this regard, recent studies showed that the recruitment of monocyte-derived alveolar macrophages in response to lung injury plays a pathogenic role in lung fibrosis and that their ablation attenuated disease progression (59, 60). Given that macrophages/fibroblasts crosstalk has been shown to contribute to lung fibrosis progression (61, 62), our findings suggest that aged lung fibroblasts may facilitate immune cell recruitment, which consequently may sustain fibroblast activation and persistence.

We previously demonstrated that epigenetic regulators play a critical role during fibroblast activation (63–65), and we identified chromobox homolog 5 (CBX5) as an epigenetic repressor that maintain IPF-derived fibroblasts in an activated state (63). Interestingly, PIM1 has been demonstrated to phosphorylate CBX3 on serine 93, a site that is also conserved in CBX5, and enhanced its capacity to bind trimethylated histone H3K9 (66). These data suggest that PIM1 may engage epigenetic regulators to perpetuate lung fibroblast activation in aging.

PIM1 has been shown to be upregulated by IL-6 through the Janus kinase–signal transducer and activator of transcription (JAK-STAT) pathway (67, 68). Beside IL-6, other cytokines and chemokines whose expression increases with aging, including IFN-α and IL-12, were shown to increase PIM1 expression (69), suggesting that aging-associated sterile inflammation underpins the increased expression of PIM1 in aged lung fibroblasts.

Immunostaining of IPF lung tissues identified high PIM1-expressing cells within fibroblastic foci, and our in vitro data demonstrate that PIM1 overexpression in normal lung fibroblasts induced BAD phosphorylation and inhibited caspase-3 activation induced by FAS-activating antibodies. These data suggest that increased PIM1 activity in IPF fibroblasts may directly promote apoptosis resistance in these cells. In line with this evidence, treatment of IPF-derived fibroblasts with the PIM1 inhibitor, AZD1208, promoted staurosporine- and FAS-activating antibody–induced caspase-3 cleavage, suggesting that targeting PIM1 in IPF may facilitate myofibroblast clearance. Evidence connecting persistent lung fibrosis to deficient apoptosis was reported in a recent study demonstrating that conditional deletion of Fas in mesenchymal cells during lung fibrosis resolution impaired apoptosis in these cells and delayed fibrosis resolution (11). Furthermore, inhibition of PIM1 has also been reported to enhance fibrosis resolution following bleomycin challenge in young mice (70), suggesting that PIM1 inhibition may represent an effective strategy to block persistent lung fibrosis and promote resolution.

We also showed that overexpression of PIM1 in normal human lung fibroblasts potentiated the transcription of profibrotic genes by TGF-β and that this effect was attenuated by NFATc1 inhibition, suggesting that the PIM1/NFATc1 axis facilitates myofibroblast differentiation. NFATc1 is a transcription factor implicated in T cell activation (34) and T cell exhaustion (71). PIM1 was shown to directly phosphorylate NFATc1, and this modification enhances its transcriptional activity (19). Our motif analysis revealed that approximately a third of all differentially expressed genes in injured aged lung fibroblasts contained binding motifs for NFATc1 and, among them, were numerous prosurvival and profibrotic genes.

Consistent with its role in regulating cell survival and proliferation, overexpression of a mutated form of NFATc1 that caused its nuclear retention perpetuated fibroblast proliferation in the absence of growth factors (72). Intriguingly, NFATc2, another NFAT family member closely related to NFATc1, has been shown to promote lung fibroblast proliferation in response to hypoxia, suggesting a potential engagement of multiple NFAT transcription factors in perpetuating lung fibroblast activation in response to different fibrogenic cues (73). Our in vitro data show that NFATc1 silencing or inhibition of its nuclear translocation in IPF fibroblasts limited PIM1 profibrotic functions and blocked TGF-β–promoted fibroblast activation, thus establishing a fibrogenic function for NFATc1 besides its involvement in fibroblast proliferation. In support of its profibrotic properties, a previous report showed that inhibition of NFATc1 by tacrolimus in mice exposed to bleomycin inhibited lung fibrosis progression (74).

Whereas we showed that inhibition of NFATc1 attenuated PIM1-promoted fibroblast activation, our work did not conclusively demonstrate that NFATc1 directly participates in PIM1-related signaling leading to fibroblast activation. More work is needed to dissect the involvement of this transcription factor as a requisite mediator of the PIM1 signaling cascade leading to lung fibroblast activation.

Our data also show that inhibition of NFATc1 in IPF-derived lung fibroblasts attenuated the expression of prosurvival genes induced by PDGF, including BIRC5 and FOXM1; however, this effect was not sufficient to sensitize these cells to apoptosis. In fact, in our hands, inhibition of NFATc1 alone failed to promote caspase-3 cleavage and did not sensitize IPF-derived lung fibroblasts to FAS activation, suggesting that NFATc1’s profibrotic function may be limited to the capacity of this transcription factor to support PIM1-promoted myofibroblast differentiation rather than apoptosis resistance. Surprisingly NFATc1 was shown to upregulate decoy receptor 3 in IPF-derived lung fibroblasts, and this mechanism was shown to confer resistance to FasL-induced apoptosis in vitro (35). These different experimental outcomes may be due to multiple factors such as intrinsic biological variability between patient-derived lung fibroblasts, different lung sampling strategies, dose of inhibitors used, and culture conditions. Future studies that will take in consideration these experimental variables will be needed to further elucidate the contribution of NFATc1 and its transcriptional program to lung fibroblast survival.

Recent scRNA-Seq analysis of whole lung derived from bleomycin-treated Col1a1-GFP^+^ transgenic mice, as well analysis of IPF lungs, identified a distinct high collagen–expressing fibroblast population defined by the expression of the fibroblast gene markers *Cthrc1*, *Postn*, and *Col3a1* (7). Consistently, our RNA-Seq analysis, which was also carried out on Col1a1-GFP mice, identified elevated expression of these 3 genes in aged lung fibroblasts following bleomycin challenge, suggesting that aging may contribute to the accumulation of this pathogenic fibroblast population in the fibrotic lungs. Additionally, our motif analysis identified NFATc1 binding motifs in the promoter of numerous genes whose expression was enriched in aged lung fibroblasts, as well as in the *Cthrc1*^+^ pathogenic fibroblast population. Notably, following an interrogation of scRNA-Seq data sets from IPF lungs, we discovered that both PIM1 and NFATc1 expression was highly enriched in the *CTHRC1*^+^ fibroblast population in IPF lungs but not in normal control lungs. Taken together, these findings strongly suggest that activation of the PIM1/NFATc1 signaling pathway in aging and in IPF lungs may perpetuate pathogenic signaling pathways to sustain fibroblast activation.

Ex vivo cultures of normal human lung explants have been demonstrated to be a useful model to study lung fibrogenesis in response to profibrotic cues (75, 76). Here, we cultured freshly isolated IPF-derived lung explants ex vivo and demonstrated that inhibiting PIM1 in these tissues resulted in reduced profibrotic gene expression and collagen secretion. Intriguingly, we found that NFATc1 inhibition by VIVIT, but not by tacrolimus, partially limited TGF-β–induced profibrotic gene expression and collagen secretion, suggesting that blocking NFATc1 activity alone in a multifaceted and heterogenic disease setting may limit, at least in part, its antifibrotic potential. Although both VIVIT and tacrolimus can inhibit NFATc1 activation, they differ in their mechanism of action. VIVIT inhibits NFATc1 dephosphorylation and nuclear translocation by specifically blocking the NFATc1/calcineurin interaction without compromising calcineurin phosphatase activity (46). In contrast, tacrolimus inhibits calcineurin enzymatic activity directly, thereby affecting downstream signaling pathways beyond NFATc1 nuclear translocation (77). These data suggest that a more selective NFATc1 inhibition strategy is needed to interrupt the profibrotic activity of NFATc1 without disturbing other signaling pathways that would otherwise compromise its therapeutic function. In addition, given the complexity and the multicellular nature of the IPF lung explants, inhibiting PIM1 in this context may impinge on multiple pathways and cell types, thus reinforcing its antifibrotic activity; inhibiting NFATc1 alone may not be as effective.

In conclusion, our study identifies the PIM1 signaling pathway as a key contributor of the persistent fibroblast activation observed in aged mice after bleomycin. PIM1 and its downstream effector NFATc1 are enriched in the fibrotic foci of IPF lungs and their inhibition in IPF-derived lung fibroblasts in vitro and in human IPF lung explants ex vivo attenuated fibrogenic activation. Collectively, our study implicates the PIM1 signaling pathway as a potentially novel target for the treatment of IPF.

## Methods

### Procurement of human IPF cells and lung tissues.

IPF cells and lung tissue from patients with IPF and from nonfibrotic healthy controls were generated in-house. Diagnoses of patients with IPF were established by clinical pathologic criteria and confirmed by multidisciplinary consensus conference. All IPF tissues were derived from explanted lungs obtained at the time of transplantation. Normal control lungs were obtained from deceased donors (Gift of Life, Michigan) whose lungs were deemed unsuitable for transplant.

### Cell culture and treatment.

All primary lung fibroblasts were obtained as mentioned above and maintained in MEM (Corning) containing 10% FBS (Gibco), 100 U/mL penicillin (Gibco), and 100 μg/mL streptomycin (Gibco) and used between passages 3 and 8. Cells were kept in a humidified incubator at 37°C with 5% CO_2_. Cells were treated with 2 ng/mL of TGF-β (PeproTech) or 50 ng/mL of PDGF-BB (PeproTech) in serum-free medium for the indicated time. AZD1208 (Tocris) was used at 10 μM at the indicated time. Apoptosis was induced by staurosporine (3 nM) or FAS-activating antibody, clone CH11 (catalog 05-201) (MilliporeSigma) at a concentration of 0.5 μg/mL for 24 hours.

### Lentivirus transduction of primary human lung fibroblasts.

The lentiviral vector pLV-EGFP-CMV encoding mCherry or human PIM1 was bought packaged in viral particles from VectorBuilder. Normal human lung fibroblasts at passage 3 were transduced at a multiplicity of infection of 3 with control or PIM1 carrying lentivirus in MEM (Corning) containing 10% FBS (Gibco), 100 U/mL penicillin (Gibco), 100 μg/mL streptomycin (Gibco), and 5 μg/mL polybrene (MilliporeSigma) for 48 hours, after which the cells were expanded and sorted by FACS for GFP^+^ cells. After sorting, cells were expanded further and used for experiments.

### Bleomycin model of lung fibrosis.

Col1α1-GFP transgenic mice on a FVB background were provided by Derek Radisky (Mayo Clinic, Jacksonville, Florida, USA) and generated as previously described (78). Two-month-old (young) or 18-month-old (aged) male mice were anesthetized with 90–120 mg/kg ketamine and 10 mg/kg xylazine. Mice were treated with 1.2 U/kg bleomycin (APP Pharmaceutical) or saline delivered intratracheally using a MicroSprayer (Penn-Century).

### FACS.

Single cell suspensions were isolated from mouse lungs 30 days after bleomycin or vehicle delivery and sorted by FACS as described in the Supplemental Methods.

### RNA-Seq analysis and gene pathway analysis.

RNA-Seq was done as previously described (9). Genes with an average raw gene count less than 25 were excluded from the differential expression analysis. Differentially expressed genes of sorted Col1a1-GFP^+^ fibroblasts between 2 groups were identified using Smyth’s moderated *t* test and Benjamini-Hochberg procedure for adjusted *P* value (FDR). Genes with an FDR of less than 0.10 and a fold change greater than 1.5 were defined as being differentially expressed. Heatmaps were created using by Prism 8 (GraphPad Software) based on *Z* scores of reads per kilobase of transcript, per million mapped reads (RPKM). Gene pathway analyses were performed using differentially expressed genes with IPA software (QIAGEN). RNA-Seq data from lung fibroblast isolated from young (GSE161322, https://www.ncbi.nlm.nih.gov/geo/query/acc.cgi?acc=GSE161322) and aged (GSE191208, https://www.ncbi.nlm.nih.gov/geo/query/acc.cgi?acc=GSE191208) mice are available on the Gene Expression Omnibus repository.

### scRNA-Seq analysis of publicly available data sets.

Publicly availably human scRNA-Seq data sets from Tsukui et al. (7) (GSE132771) were downloaded from the BioTuring repository and analyzed using the BioTuring Browser (79) (BioTuring). All cell type annotations were those of Tsukui et al. (7). Only normal and IPF cells were included in the analysis. All scRNA data are displayed as UMAP plots.

### IHC.

Formalin-fixed paraffin-embedded blocks of normal and IPF lung tissues were generated in-house. Paraffin blocks were sectioned at 5 μm thickness and mounted on Superfrost Plus slides (Thermo Fisher Scientific). The sections were deparaffinized in histoclear and rehydrated through graded ethanol. Antigen retrieval was performed by incubating slides for 20 minutes in citrate buffer (pH 6.0) at 98°C. After cooling, endogenous peroxidases were blocked with BLOXALL (Vector Laboratories) for 10 minutes and then incubated with 2.5% horse serum to block nonspecific binding for 30 minutes at room temperature. Stainings were performed by using the ImmPRESS HRP PLUS polymer kit (Vector Laboratories) following the manufacturer protocol. Human lung tissues were stained with NFATc1 antibody (NB10056732) at 1:200. (Novus biologicals) or PIM1 antibody at 1:200 dilution (MA5-35347, Thermo Fisher Scientific). Slides were then counterstained with hematoxylin and coverslipped.

### Immunofluorescence.

IPF lung fibroblasts were grown on coverslips and treated with DMSO as vehicle or 2 ng/mL of TGF-β alone or in combination with 10 μM of AZD1208 for 24 hours in serum-free MEM. Following treatment, cells were stained as described in the Supplemental Methods.

### RNA interference.

RNA interference was performed using Lipofectamine RNAiMAX reagent (Thermo Fisher Scientific) as described in the Supplemental Methods.

### Reverse transcription and real-time PCR.

Total RNA was isolated using Quick-RNA Miniprep Kit (Zymo Research) following manufacturer protocol. Following quantification of RNA concentration, equal mass of RNA was used for reverse transcription using the High-Capacity cDNA Reverse Transcription Kit (Thermo Fisher Scientific) following manufacturer protocol. qPCR was performed using PowerUp SYBR Green Master Mix (Applied Biosystems) with specific primers. Primer sequences are available in Supplemental Table 1.

### Protein extraction and Western blotting.

Western blot analysis of protein lysates was performed as described in the Supplemental Methods. Densitometry analysis of Western blots is shown in Supplemental Figure 4.

### ECM deposition assay.

Cells transduced with control or PIM1 lentivirus were grown in clear-bottom 96-well plates with MEM containing 2% FBS, 20 μg/mL ascorbic acid, and 20 μg/L Copper (II) Sulfate. After 3 days, cellular ECM deposition was measured as described in the Supplemental Methods.

### Organotypic human IPF lung cultures.

Lung tissue from patients with IPF were generated in-house as mentioned above. Upon arrival, lung tissues were cut into thin slices and punched into small discs using a biopsy puncher. IPF lung explants were maintained in complete MEM containing 10% FBS, 100 U/mL penicillin, 100 μg/mL streptomycin, and 1 μg/mL amphotericin B. Lung explants were kept in a humidified incubator at 37°C with 5% CO_2_. The IPF lung explants were cultured with or without 10 ng/mL TGF-β and in combination with or without 10 μM of AZD1208 in complete medium for 5 days. IPF lung explants were also cultured with or without 10 ng/mL TGF-β and in combination with or without 5 μM of VIVIT or 10 μM of tacrolimus in complete medium for 5 days. Treatment media was replaced every 48 hours.

### Statistics.

Individual data points are shown in all plots and represent data from independent mice or biological replicates from cell culture experiments. Comparison between 2 groups was calculated using 2-tailed Student’s *t* test, and comparison between multiple groups was calculated using 1-way ANOVA with Holm-Šidák post hoc test. All statistical analyses were performed using GraphPad Prism 9.3.0, with *P* values labeled on graphs. Statistical significance was set at *P* < 0.05.

### Study approval.

All patient samples were obtained with informed consent and were approved by the University of Michigan IRB (no. HUM00105694). All mouse experiments were carried out in accordance with the Boston University and Mayo Clinic IACUC and followed the ARRIVE guidelines.

## Author contributions

TXP and GL conceived and designed the project. TXP, JL, JG, NC, JAM, DLJ, and QT performed experiments. TXP, SKH, DJT, and GL interpreted the data. TXP and GL wrote the manuscript. All authors edited and approved the manuscript.

## Supplementary Material

Supplemental data

## Version 1 02/15/2022

In-Press Preview

## Version 2 03/22/2022

Electronic publication
