# Supplementary material for: Transcriptional analysis of lung fibroblasts identifies PIM1 signaling as a driver of aging-associated persistent fibrosis
Source: JCI Insight. 2022 Mar 22;7(6):e153672. doi: 10.1172/jci.insight.153672 (PMC8986080; doi:10.1172/jci.insight.153672)
Supplement: Supplemental data [file jciinsight-7-153672-s016.pdf]

Supplemental Figures

Supplemental Figure S1

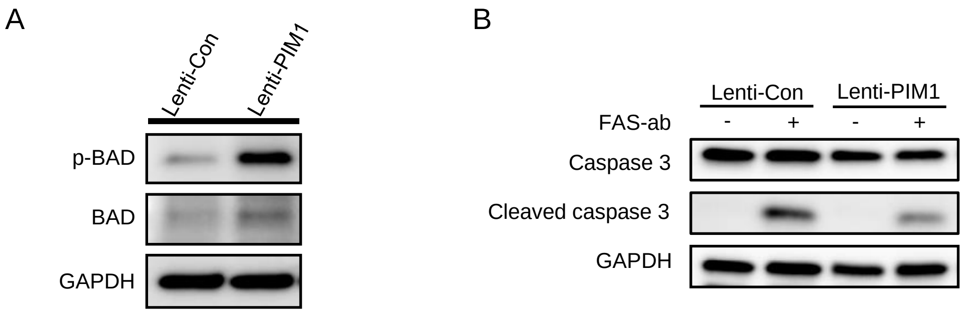

Supplement Fig.S1. **PIM1 overexpression in normal human lung fibroblasts induces BAD phosphorylation and inhibits Fas-Ab-induced caspase-3 activation**

A. Western blot showing incresed BAD phosphorylation (p-BAD) in PIM1-overexpressing human lung fibroblasts. Shown is a representative blot of n=3 independent experiments. **B**. Western blotting showing reduced Fas-ab-induced caspase-3 cleavage in PIM1-overexpressing lung fibroblasts compared to control cells. Shown is a representative blot of n=2 independent experiments.

Supplemental Figure S2

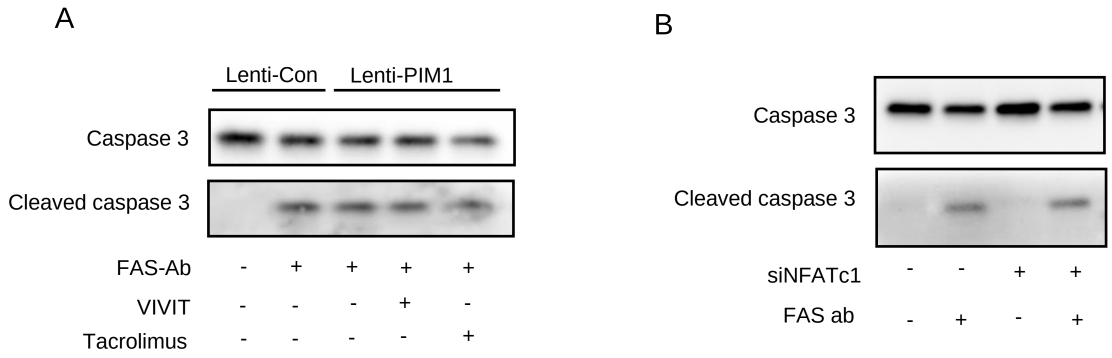

Supplement Fig.S2. **Inhibition of NFATc1 failed to promote caspase-3 cleavage and does not sensitize IPF-derived lung fibroblasts to FAS activation**

A. Western blot showing reduced cleaved caspase-3 in PIM1 overexpressing cells treated with NFATc1 inhibitors, VIVIT (5 µM) or Tacrolimus (1 µM), for 24 h followed by additional 24 h treatment with or without FAS-activaing ab (500 ng/mL). Blots shown are representative of n=2 independent experiments. **B.** Western blot showing no change in cleaved caspase-3 in IPF-derived lung fibroblasts silenced for NFATc1 for 48 h and then treated with FAS-activating antibody for additional 24 h, n=1.

Supplement Figure S3

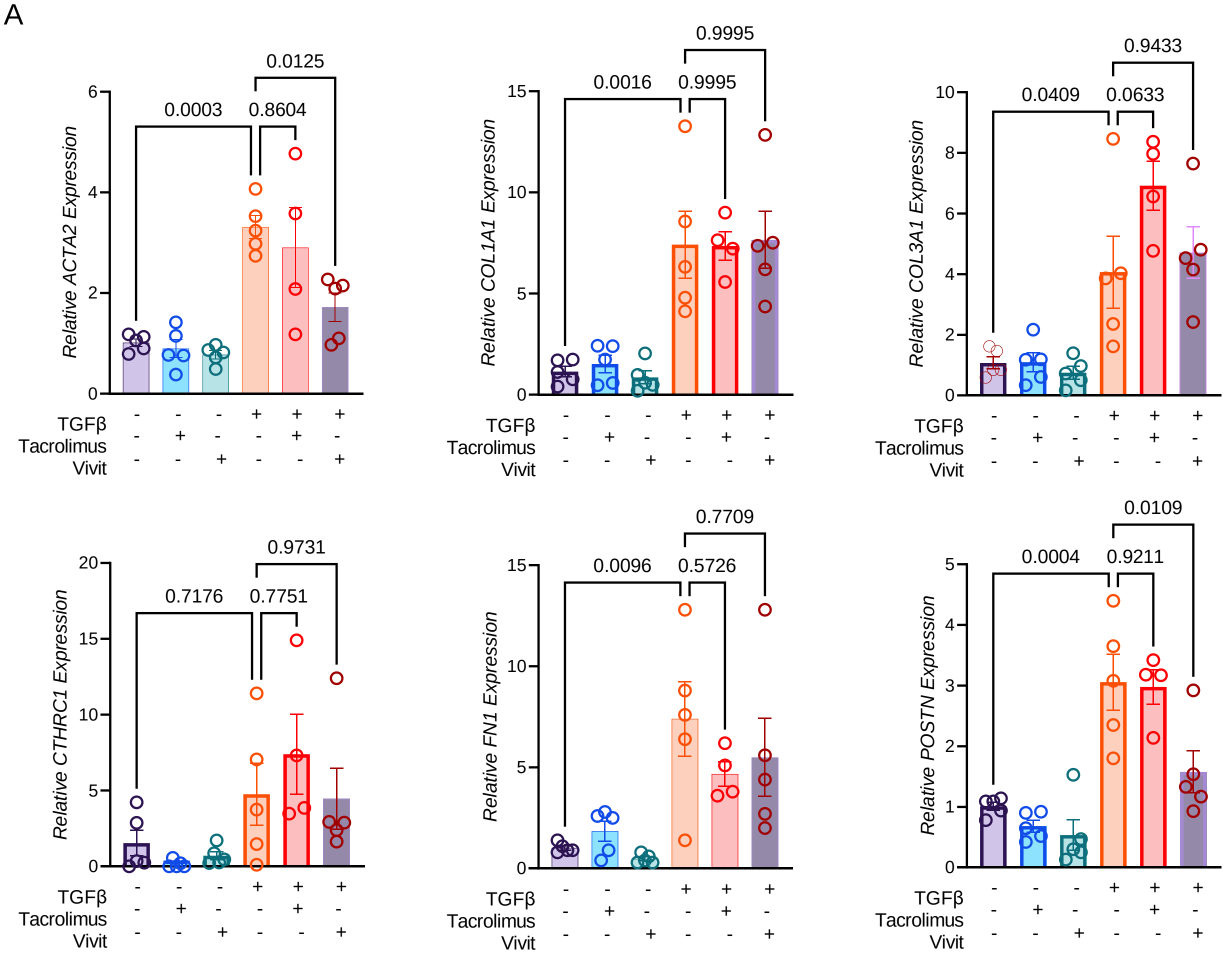

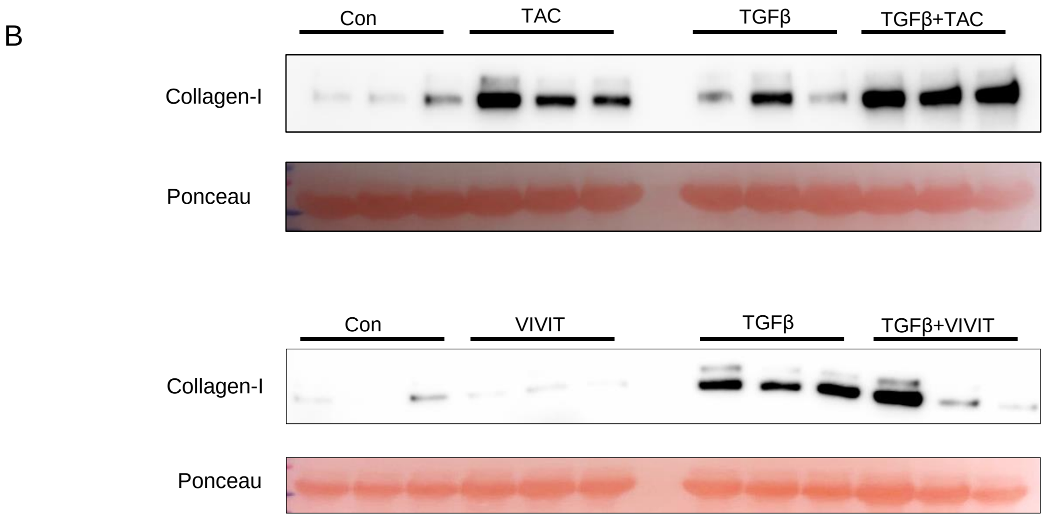

Supplemental Fig. S3. NFATc1 inhibition by VIVIT, but not by tacrolimus, reduces pro-fibrotic gene expression and collagen secretion in organotypic IPF lung cultures *ex vivo*

A. qPCR analysis of ECM gene expression in IPF lung explants treated with 5 µM of VIVIT or 10 µM of Tacrolimus in the presence or absence of 10 ng/mL of TGFβ for 5 days, n ≥ 4 IPF lung explants. Data are shown as mean ± SEM. P-value were calculated using one-way ANOVA with Holm-Sidak post-hoc test. **B.** Soluble collagen secreted from IPF lungs treated as described in (A) into the media was evaluated by Western blot analysis. Each lane contained equal volume of conditioned medium of different lung sections obtained from single IPF lung explants.

Supplement Figure S4

Figure 4D

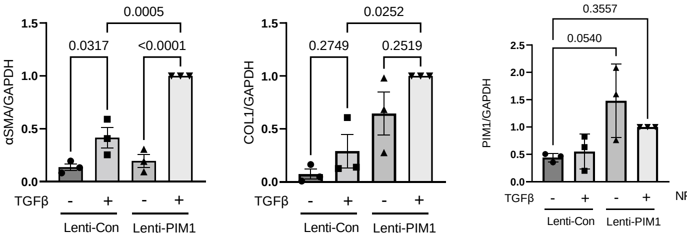

Figure 4G

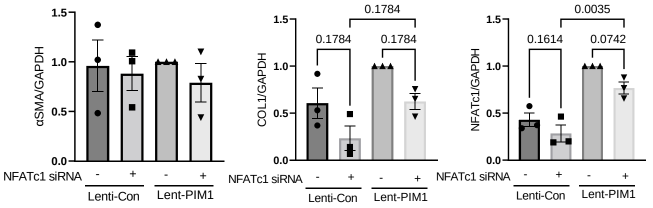

Figure 4I

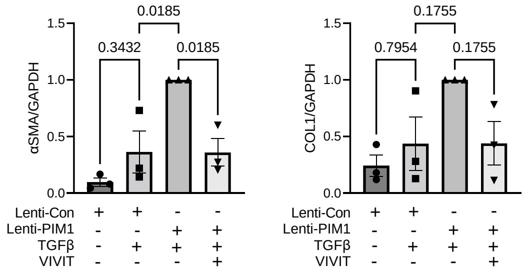

Figure 4J

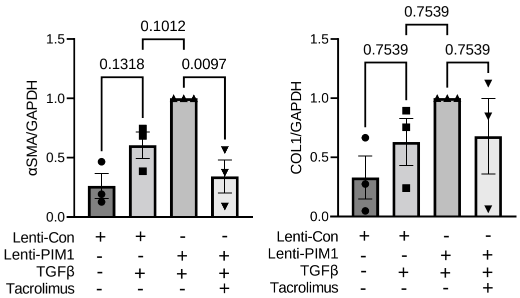

Figure 5B

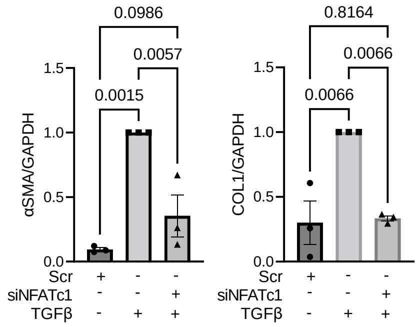

Figure 5D

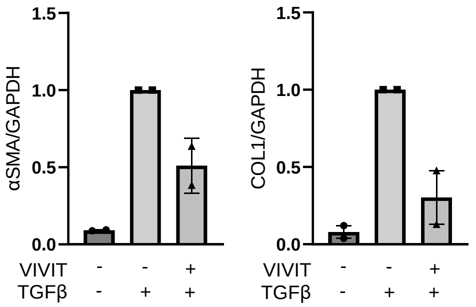

Figure 5F

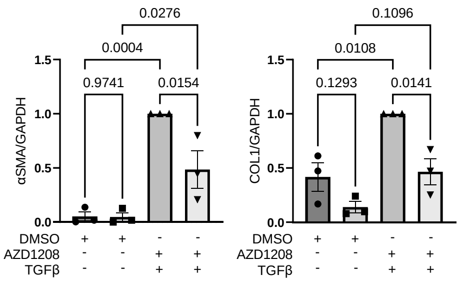

Figure 6B

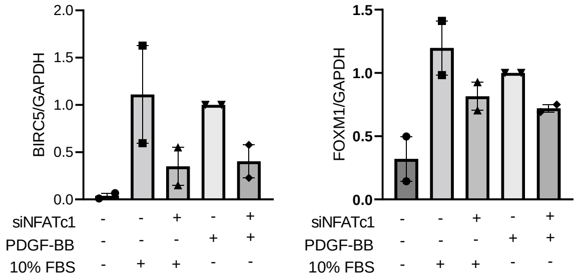

Figure 6E

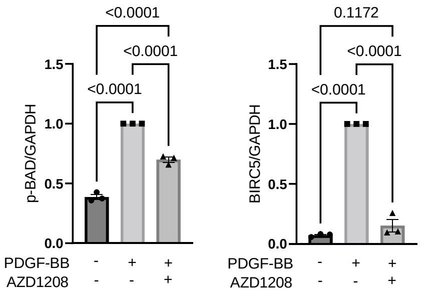

Supplemental Fig. S4. Quantification of Western blots

Densitometry analysis of key western blots from which representative blots were selected and shown in the figures. Data are shown as mean ± SEM. P-value were calculated using one-way ANOVA with Holm-Sidak post-hoc test.

Supplemental Table 1. List of primers used for quantitative PCR

Primers	Forward (5’-3’)	Reverse (5’-3’)
ACTA2	AAAAGACAGCTACGTGGGTGA	GCCATGTTCTATCGGGTA CTTC
COL1A1	GAGGGCCAAGACGAAGACATC	CAGATCACGTCATCGCACAAC
COL3A1	GGAGCTGGCTACTTCTCGC	GGGAACATCCTCCTTCAACAG
FN1	CGGTGGCTGTCAGTCAAAG	AAACCTCGGCTTCCTCCATAA
CTHRC1	CAATGGCATTCCGGGTACAC	GTACACTCCGCAATTTTCCCAA
POSTN	GCTATTCTGACGCCTCAAAACT	AGCCTCATTACTCGGTGCAAA
NFATC1	CACCGCATCACAGGGAAGAC	GCACAGTCAATGACGGCTC
FOXM1	CGTCGGCCACTGATTCTCAAA	GGCAGGGGATCTCTTAGGTTC
BIRC5	AGGACCACCGCATCTCTACAT	AAGTCTGGCTCGTTCTCAGTG
PLK1	CACCAGCACGTCGTAGGATTC	CCGTAGGTAGTATCGGGCCTC
RPLP0	AGCCCAGAACACTGGTCTC	ACTCAGGATTTCAATGGTGCC

Supplemental Methods

Fluorescence-activated cell sorting (FACS)

Mice were anaesthetized with ketamine/xylazine and perfused via the left ventricle with cold PBS. The lungs were immediately harvested and minced with a razor blade in a 100 mm petri dish in cold MEM medium containing 0.2 mg/mL Liberase DL and 100 U/mL DNase I (Roche, Indianapolis, IN, USA). The mixture was transferred to 15 mL tubes and incubated at 37 °C for 40 min under continuous rotation. Digestion was inactivated with MEM containing 10% FBS and the resulting suspension was passed through a 40 µm cell strainer (ThermoFisher Scientific, Waltham, MA, USA) to remove debris. Cells were then centrifuged at 300 g for 10 min at 4°C and then resuspended in 2 mL of red blood cell lysis buffer (ThermoFisher Scientific, Waltham, MA, USA) for 90 seconds to remove red blood cells and then diluted in 9 mL PBS. Cells were then centrifuged (300 g, 10 mins, 4°C) and resuspended in 0.2 mL of FACS buffer (1% BSA, 0.5 mM EDTA pH 7.4 in PBS). The single cell suspension was incubated with anti-CD45:PerCp-Cy5.5 (1:200, Biolegend, San Diego, CA, USA, Cat# 103132), anti-CD31:PE (1:200, Biolegend, San Diego, CA, USA, Cat#102408), anti-EpCAM:APC (1:200,Biolegend, San Diego, CA, USA, Cat#118214) antibodies and DAPI (1:1000, Biolegend, San Diego, CA, USA, Cat#422801) for 30 min on ice. After incubation, cells were washed with ice-cold FACS buffer and resuspended in 1 ml of FACS buffer. FACS sorting was conducted using a BD FACS Aria II (BD Biosciences, San Jose, CA, USA) as previously described (9, 16). Cells were sorted directly into RNeasy micro kit RLT buffer (Qiagen, MD, USA). Total mRNA was isolated using RNeasy micro kit, followed by Nanodrop concentration and purity analysis.

Immunofluorescence

IPF lung fibroblasts were grown on coverslips and treated with DMSO as vehicle or 2 ng/mL of TGFβ alone or in combination with 10 µM of AZD1208 for 24 h in serum-free MEM media.

Following treatment, the cells were fixed with 10% formalin for 10 minutes at room temperature and then washed three times with ice-cold PBS. The cells were then permeabilized with 0.1% Triton X-100 for 5 mins. Following three washes with PBS, cells were incubated with Intercept Blocking Buffer (Li-Cor, Linocln, NE, USA) for 30 mins and then stained with αSMA at 1:200 (A2547) (MilliporeSigma, Burlington, MA, USA) overnight. Next day, the cells were washed 3 times with PBS and then incubated with secondary antibodies conjugated with PE diluted at 1:1000 and containing DAPI stain (1 mg/mL; dilution 1:1000) for 30 mins. Following three washes with PBS, the coverslip containing cells were mounted onto slides using Aqua Poly mounting medium (Polysciences Inc, Warrington, PA, USA) and then imaged using a confocal microscope (Olympus FluoView FV10i).

RNA interference

RNA interference was performed with siGENOME Non-Targeting Control siRNA Pool #1 (D-001206-13-05) or SMARTpool ON-TARGETplus Human *NFATC1* siRNA (L-003605-00-0005) or siGENOME Human *PIM1* siRNA (D-003923-01-0002) (Horizon Discovery, Waterbeach, UK) by using Lipofectamine RNAiMAX reagent (ThermoFisher Scientific, Waltham, MA, USA) as previously described (17).

Reverse transcription and Real-time PCR

Total RNA was isolated using Quick-RNA Miniprep Kit (Zymo Research, Irvine, CA, USA) following manufacturer's protocol. Following quantification of RNA concentration, equal mass of RNA was used for reverse transcription using the High-Capacity cDNA Reverse Transcription Kit (ThermoFisher Scientific, Waltham, MA, USA) following manufacturer's protocol. qPCR was performed using PowerUp SYBR Green Master Mix (Applied Biosystems, Foster City, CA, USA) with specific primers. All primer sequences are available upon request.

Protein extraction and Western Blotting

Cell protein were extracted using RIPA lysis buffer (ThermoFisher Scientific, Waltham, MA, USA). Protein concentration was determined using the Pierce BCA Protein Assay (ThermoFisher Scientific, Waltham, MA). Equal amount of protein from each sample or equal volume of conditioned medium were loaded on a 4-15% polyacrylamide Mini-PROTEAN precast gel (Bio-Rad, Hercules, CA, USA) and separated by electrophoresis. The protein were then transferred onto PVDF membrane, blocked with 5% non-fat dry milk, and incubated with primary antibodies overnight at 4°C. Western blotting analysis of cell lysates was performed using the following antibodies against: FOXM1(#5436), GAPDH (#5174), COL1A1 (#72026), CASPASE 3 (#9662), Phospho-BAD (Ser112) (#5284), BAD (#9292) (Cell Signaling, Danvers, MA, USA,), BIRC5 (NB500-201SS), COL I (NB600-408) (Novus Biologicals, Littleton, CO, USA), αSMA (A2547) (MilliporeSigma, Burlington, MA, USA), PIM1(MA5-35347) (Thermo Fisher Scientific, Waltham, MA, USA). Blots were then washed and incubated with appropriate IgG-HRP-conjugated antibodies for 1 hour at room temperature. Bands were visualized by using Super Signal West Pico Plus or Super Signal West Femto (Thermo Fisher Scientific, Waltham, MA, USA) and ChemiDoc (Bio-Rad, Hercules, CA, USA) according to the manufacturer's protocol. Western blot images were quantified using Image Lab 6.0.1 (Bio-Rad, Hercules, CA, USA).

ECM Deposition Assay

Cells transduced with control or PIM1 lentivirus were grown in clear-bottom 96-well plates with MEM media containing 2% FBS, 20 µg/mL ascorbic acid, and 20 µg/L Copper (II) Sulfate. After 3 days, each well was de-cellularized with 20 mM NH_4OH for 10 mins and then washed with PBS three times. Cell matrix in each well were then fixed with 10% formalin for 15 mins at room temperature. After the cells were washed with PBS, they were incubated with Intercept Blocking Buffer (Li-Cor Biosciences, Lincoln, NE, USA) to block unspecific binding for 60 mins and incubated with primary Collagen I (Novus NB600-408) antibodies at 1:600 dilution,

overnight, and at 4°C. The next day, the cells were washed with PBS with 0.1% (v/v) Tween-20 and then stained with secondary anti-rabbit IgG IRDye 800 antibody at 1:1000 dilution for 1 h and then washed before read. The microplates were scanned with the Odyssey CLx Infrared Imaging System (Li-Cor,Biosciences, Lincoln, NE, USA) and the integrated fluorescence intensities were acquired using Image Studio software version 3.0 (Li-Cor Biosciences, Lincoln, NE).

Immunohistochemistry

Formalin-fixed paraffin-embbed blocks of normal and IPF lung tissues were obtained from Dr. Steven K. Huang at the University of Michigan. Paraffin blocks were sectioned at 5 µm thickness and mounted on Superfrost Plus slides (Thermo Fisher Scientific, Waltham, MA, USA). The sections were deparaffinzed in histoclear and rehydrated through graded ethanol. Antigen retreival was performed by incubating slides for 20 mins in citrate bufer (pH 6.0) at 98°C. After cooling, endogenous peroxidases were blocked with BLOXALL (Vector Laboratories, Peterborough, UK) for 10 mins and then incubated with 2.5% horse serum to block nonspecific binding for 30 mins at room temperature. Stainings were performed by using the ImmPRESS HRP PLUS polymer kit (Vector Laboratories) following the manufacturerer's protocol. Human lung tissues were stained with NFATc1 antibody (NB10056732) at 1:200. (Novus biologicals, Littleton, CO, USA) or PIM1 antibody at 1:200 dilution (MA5-35347) (Thermo Fisher Scientific, Waltham, MA, USA). Slides were then counterstained with hematoxylin and coverslipped.
